# Genetic Control of Organ Shape and Tissue Polarity

**DOI:** 10.1371/journal.pbio.1000537

**Published:** 2010-11-09

**Authors:** Amelia A. Green, J. Richard Kennaway, Andrew I. Hanna, J. Andrew Bangham, Enrico Coen

**Affiliations:** 1 Department of Cell and Developmental Biology, John Innes Centre, Norwich, United Kingdom; 2 University of East Anglia, School of Computing Sciences, Norwich, United Kingdom; University of York, United Kingdom

## Abstract

The mechanisms by which genes control organ shape are poorly understood. In principle, genes may control shape by modifying local rates and/or orientations of deformation. Distinguishing between these possibilities has been difficult because of interactions between patterns, orientations, and mechanical constraints during growth. Here we show how a combination of growth analysis, molecular genetics, and modelling can be used to dissect the factors contributing to shape. Using the Snapdragon (*Antirrhinum*) flower as an example, we show how shape development reflects local rates and orientations of tissue growth that vary spatially and temporally to form a dynamic growth field. This growth field is under the control of several dorsoventral genes that influence flower shape. The action of these genes can be modelled by assuming they modulate specified growth rates parallel or perpendicular to local orientations, established by a few key organisers of tissue polarity. Models in which dorsoventral genes only influence specified growth rates do not fully account for the observed growth fields and shapes. However, the data can be readily explained by a model in which dorsoventral genes also modify organisers of tissue polarity. In particular, genetic control of tissue polarity organisers at ventral petal junctions and distal boundaries allows both the shape and growth field of the flower to be accounted for in wild type and mutants. The results suggest that genetic control of tissue polarity organisers has played a key role in the development and evolution of shape.

## Abstract 2

A combination of experimental analysis and mathematical modelling shows how the genetic control of tissue polarity plays a fundamental role in the development and evolution of form.

## Author Summary

Genes are known to control the shape of biological structures, like flowers, hearts, and limbs, yet how they do this is poorly understood. A working hypothesis is that genes control shape by modulating local rates at which growing tissue deforms. Evaluating this idea has been difficult, however, because of the dynamic interactions that occur within growing and deforming tissue. To address this problem, we used a combination of experimental and mathematical modelling approaches to study how genes control shape in the Snapdragon flower. This system has the advantages of having well defined genes that influence shape and being accessible to growth analysis. We first tried to explain the experimental data with a model in which genes influence local rates of tissue growth. While this model could capture many aspects of flower development, it failed to account for some key features. These could be most readily explained if genes also affect an internal field of orientations along which growth is directed, established by organisers of tissue polarity. Our analysis therefore revealed a previously unsuspected role of shape genes in the control of tissue polarity, highlighting the importance of this process for the development and evolution of tissue forms.

## Introduction

Changes in tissue shape during development may involve growth, cell rearrangements, or a combination of the two. In the case of plants, where cells are held in place by their walls, shape changes arise purely through differential growth [1]–[3]. Thus, plants provide a simplified system for studying the genetic control of shape. Typically, plant tissues do not grow equally in all directions but preferentially along particular orientations. In principle, genes may therefore influence plant shape by modifying rates and/or orientations of growth [4]. Separating these two aspects is critical for understanding how genes control shape, yet it is often difficult to achieve because of the mechanical constraints that arise from tissue connectedness. For example, enhanced growth of one tissue region may lead to bending and distortions of nearby connected tissue. These deformations will influence local orientations, which in turn influence growth and so further deformations [5]. One way to dissect these interactions is to incorporate proposed gene mechanisms within models of growing tissue. By comparing the output of such models with experimental data on gene expression patterns, growth dynamics, and shape in wild-type and mutant backgrounds, various hypotheses for how genes control shape can be evaluated. Here we apply this approach to the *Antirrhinum* (Snapdragon) corolla, which has the advantage of representing a complex shape for which molecular genetic and growth data can be readily obtained.

**Figure 1:**
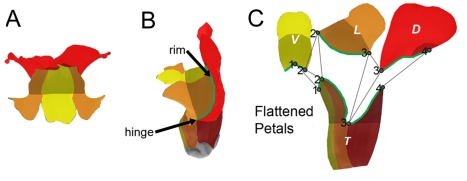
Shape of the wild-type *Antirrhinum* flower. (A) Face view. Dorsal lobes are coloured red, lateral lobes orange, ventral lobe yellow. Lip region is shaded darker. (B) Side view. Dorsal tube is dark red, lateral tube dark orange, ventral tube dark yellow. The rim is traced in green. (C) Dissected, flattened petal parts: dorsal (D), lateral (L), and ventral (V) lobes and half of the tube (T) cut along the dorsal and ventral midlines of the flower. Petal parts coloured as in (A) and (B). Palate region of the tube is shaded darker. Numbered dots show connecting positions in the un-dissected corolla.

Wild-type *Antirrhinum* flowers are bilaterally symmetrical, having two dorsal petals, two lateral petals, and one ventral petal (Figure 1). The petals are united for part of their length to form a corolla with a proximal tube and five distal lobes. The tube forms a chamber that is held shut by tightly apposing upper and lower petals at its distal rim. Floral asymmetry is established by the dorsoventral genes *CYCLOIDEA* (*CYC*), *DICHOTOMA* (*DICH*), *RADIALIS* (*RAD*), and *DIVARICATA* (*DIV*). *CYC* and *DICH* code for TCP transcription factors that are expressed in the dorsal domain of the early corolla [6]. Mutants that lack *CYC* and *DICH* have radially symmetrical flowers in which all petals resemble the ventral petal of wild type. *CYC* and *DICH* activate *RAD*, which encodes a small MYB-domain protein, which in turn antagonises the action of another MYB-domain protein, DIV, restricting its activity to the ventral and lateral domains [7]–[10]. The combinatorial effects of the various genes on the final shape of each region of the corolla have been defined through shape analysis of multiple different mutant and over-expression backgrounds (the topic of the accompanying article [11]).

Growth and development of the *Antirrhinum* corolla has been studied using a combination of scanning electron microscopy [12] and computational clonal analysis [13]. Clonal analysis utilises a temperature-sensitive transposon [14] that can be induced to excise from a flower pigmentation gene at defined stages of development. By comparing large numbers of clones induced at a range of stages, it is possible to compute rates and orientations of growth of the dorsal lobe. The results suggest that dorsal lobe shape depends largely upon how growth orientations are coordinated and maintained in the petal. This type of analysis has so far been restricted to a flattened dorsal lobe, so it is unclear how genes control growth of the entire corolla, yielding the complex 3-D flower form.

Here we show that the corolla undergoes a series of shape changes in 3-D, captured using optical projection tomography (OPT) [15]–[17]. Computational clonal analysis shows that these morphogenetic events are associated with a growth field that changes as the flower develops. Flower shape and the growth field are both altered in reproducible ways in dorsoventral mutants. These observations can be partially accounted for by models in which dorsoventral genes modulate specified growth rates along orientations specified by two organisers of tissue polarity, located at the proximal and distal boundaries of the corolla. The organisers anchor the pattern of tissue polarity and thus determine the principal orientations of specified growth. However, such models do not readily account for the observed growth orientations and shape of the lower palate and dorsal lobes. These data can be much more easily explained by models in which dorsoventral genes also modulate organisers of tissue polarity. In particular, *DIV* is proposed to control the activity of an organiser of tissue polarity at an internal boundary between ventral and lateral petals, and *DICH* modulates distal organiser activity. With these additional assumptions, the models generate growth fields and 3-D shapes in wild-type and mutant backgrounds that match observed data. Thus, our combined experimental and modelling approach suggests that genes control tissue shape not only by modulating specified growth rates but also by influencing organisers of tissue polarity and orientations of growth. We propose that effects on tissue polarity have played a key role in the evolution of shapes, such as the closed Snapdragon flower, and thus provide a general mechanism for the generation of complex forms.

## Results

### Key Morphogenetic Changes during Corolla Development

**Figure 2:**
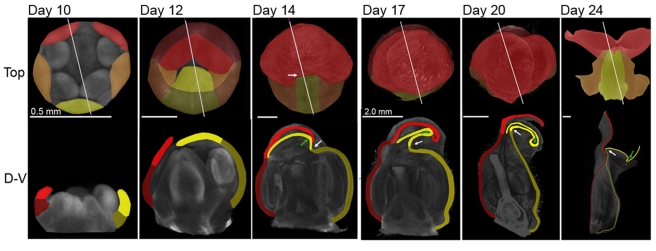
*Antirrhinum* corolla morphogenesis. Images and sections from OPT scans of *Antirrhinum* flowers (with sepals removed) at a series of developmental stages (day 10–20 after flower initiation). A mature flower was also sectioned (day 24). Each flower is shown in face view (Top). Dorsal lobes are coloured bright red, dorsal tube dark red, lateral lobes bright orange, lateral tube dark orange, ventral lobe bright yellow, and ventral tube dark yellow. Grey lines mark planes of virtual sections, shown below (D–V). The white arrow indicates the ventral furrow and the green arrow indicates a ventral fold in the lobes (day 14–24). Initially (day 10) the corolla is almost radially symmetrical. The petals arch over to form a near spherical shell (day 12). The ventral furrow then forms (day 14). At later stages the ventral tube stretches out and straightens (day 17–24). Eventually the dorsal and ventral parts of the rim meet to form an enclosed chamber and the lobes unfold to give an open flower (day 24). Scale bar is 0.5 mm for day 10–14 and 2 mm for day 17–24.

As a first step to understanding how dorsoventral genes determine flower shape, we defined the key morphogenetic changes during corolla development. Flower buds were imaged at a series of developmental stages using OPT [15],[17]. A range of stages viewed from the top or as longitudinal sections are shown in Figure 2 (see also Videos S1–S5). The buds were staged according to days after meristem initiation, determined by size and morphology [12]. At day 10, the corolla comprised a short cylindrical tube with five distal lobes. During the following four days, the corolla grew preferentially along the proximodistal axis and arched over the centre of the flower (Figure 2, days 12, 14). The ventral tube became strongly curved during this period and formed a fold at its boundary with the lobe, termed the rim (white arrows in Figure 2, lower panel). A further fold, in the opposite direction, also formed more distally within the ventral lobe (green arrow). During the following stages the flower continued to grow preferentially along the proximodistal axis, and the ventral tube gradually became stretched and less curved (Figure 2, days 17, 20). Towards the end of development, the flower opened through the petal lobes bending back (Figure 2, day 24). Thus, corolla morphogenesis can be divided into two broad phases: an early phase when the ventral tube becomes strongly curved and folded back at the rim, and a later phase when the ventral tube stretches out.

### Clonal Analysis and Estimation of Growth Parameters

Although OPT data give an overview of changing corolla geometry, a full description of corolla morphogenesis requires the growth for each region of the tissue sheet to be determined. Four local parameters are needed to describe the growth of a sheet in 2-D: the principal orientation of growth (*θ*), growth rate along this orientation (*K_max_*), growth rate perpendicular to this (*K_min_*), and rotation rate (ω). The values of these parameters for all regions over time define a dynamic growth tensor field [18]. We have used computational clonal analysis previously to estimate these parameters for the *Antirrhinum* dorsal petal lobe [13]. Here we extended this approach to the entire corolla.

**Figure 3:**
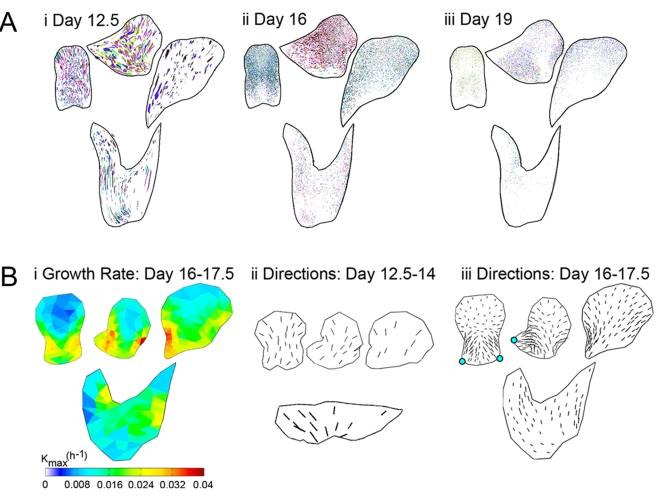
Clonal analysis of wild-type corolla. (A) Clones on dissected, flattened petals from 6 flowers induced at day 12.5 (i), day 16 (ii), and day 19 (iii), warped to a mean shape for each petal section (D, L, V, T—see Figure 1C) and overlaid, using a different colour for each flower. (B) Regional growth parameters based on clonal analysis (using grids in Figure S12). (i) Maximal growth rates, *K_max_*, for the period day 16–17.5. Rates are highest (red/yellow) along the lobe edges. (ii) Principal orientations of growth for the period day 12.5–14, shown as short lines scaled according to the value of *K_max_*. (iii) Principal orientations of growth for the day 16–17.5 interval. Cyan spots indicate foci at the ventral lobe junctions (numbered 2 in Figure 1C).

For this analysis we used a temperature sensitive transposon that can excise during development of the flower to give pigmented clones [13],[14],[19]. Clones were induced at a range of developmental stages using temperature pulses and then visualised in flattened petals from mature flowers (Figure 1C). Clones from multiple flowers were then superimposed and warped to the average petal shapes (Figure 3A [20]). Most clones induced at earlier stages were elongated and oriented in characteristic patterns, indicating that growth is anisotropic and coordinated along particular orientations (Figure 3Ai, day 12.5; Figures S1–S11). In particular, clones tended to be oriented towards foci at the junctions between petals, forming what looked like “flow” patterns.

Comparisons between average clone shapes for each region at different stages of development allow the key parameters defining the growth field of the corolla to be estimated [20]. Applying this procedure to the various petals revealed that the growth parameters varied among regions and developmental stages (Text S1A; Figures S1, S12). For example, *K_max_* varied from about 1% to 4% per hour depending on the region of the corolla during day 16–17.5 (Figure 3Bi). The principal growth orientations (*θ*) showed a striking change from early to later phases of growth. During the early phase, growth orientations were roughly parallel to the proximodistal axis (Figure 3Bii, day 12.5–14), while during the later phase, growth orientations converged at the junctions between the ventral petal boundaries and the rim (cyan spots, Figure 3Biii, day 16–17.5; see also Text S1A).

### Modelling Tissue Growth

To explore hypotheses for how dorsoventral gene activity could lead to the observed morphogenetic changes and growth fields, a modelling framework is required [21]. Previous systems for modelling tissue growth have been restricted to growth in 2-D or to growth in 3-D that is either isotropic or limited to axially symmetric structures [4],[22]–[26]. To account for more complex structures we developed a system, termed the Growing Polarised Tissue framework (GPT-framework), that can capture anisotropic growth of tissue able to deform in 3-D into arbitrary shapes [5]. With this method, growing tissue is modelled as a continuous sheet of material with two surfaces and a thickness, termed a canvas. Treating the tissue as a continuum has the advantage that overall properties of growth can be captured without having to explicitly model the millions of cells in various layers of the final corolla, saving on computational time and thus making it possible to address the complexities of tissue shape change.

Each model can be conveniently divided into three or four interacting components: (1) A setup phase during which the initial canvas and distribution of regional identities is established. This involves specifying *identity factors* that are unable to propagate through the canvas and *signalling factors* that can propagate. (2) For models incorporating gene interactions, a gene regulatory network (GRN) is defined that controls the activity of identity or signalling factors encoded by known genes. The genes within the GRN influence growth by modulating the following two components (PRN and KRN). (3) A Polarity Regulatory Network (PRN) is defined that controls the activity of various organisers from which tissue polarity information propagates. Polarity propagation is implemented through a signalling factor called POLARISER (POL), the gradient of which defines local polarity. The PRN controls production and degradation of POL at organisers that anchor the polarity. Direct gradient reading is used as a convenient way of specifying polarity at the tissue scale and should not be taken to imply that more elaborate mechanisms such as cell-cell signalling [27],[28] are not involved. (4) A growth rate (*K*) regulatory network (KRN) defines how identity or signalling factors influence *specified growth rates* in relation to local polarity. In accordance with previous studies, growth rates are expressed in relative rather than absolute terms (i.e., as a percentage of current size [29]). The specified growth rates for a region of the canvas are equivalent to the growth (strain) that would arise in the hypothetical situation in which that region grows in isolation, without the constraints of surrounding material. In practice, regions are mechanically connected through the canvas so the specified growth rates are typically not the same as the growth rates generated, termed *resultant growth rates*. For each region there are two specified growth rates in the plane of the canvas—a rate parallel to the local polarity, termed *K_par_*, and a rate perpendicular to the local polarity, *K_per_*. These specified growth rates can be further enhanced or reduced on either surface of the canvas (termed the *A* and *B* surfaces). A third specified growth rate *K_nor_* is used to define the rate of growth in tissue thickness.

The combination of components (1–4) defines a field of specified growth rates and orientations across the canvas. This specified growth field is applied to the initial canvas in the first growth time step, taking account of mechanical interactions arising from the interconnectedness of the tissue (modelled according to elasticity theory [5]). This leads to a slight deformation of the canvas (resultant growth field) and its associated identity and signalling factors. The concentrations of signalling factors are then further adjusted according to their propagation and decay rates. The deformed canvas and expression pattern provide the starting point for the next time step and the same sequence of events is iterated. Models were constrained by several bodies of experimental data: OPT images of developing buds, clonal analysis of growth in wild type and mutants, shape analysis of a range of mutants and over-expression lines, and gene expression patterns in wild-type and mutant backgrounds. The aim of the modelling was not to capture every experimental detail but the overall trends observed.

### Model for the *cyc dich div* Ground State

**Figure 4:**
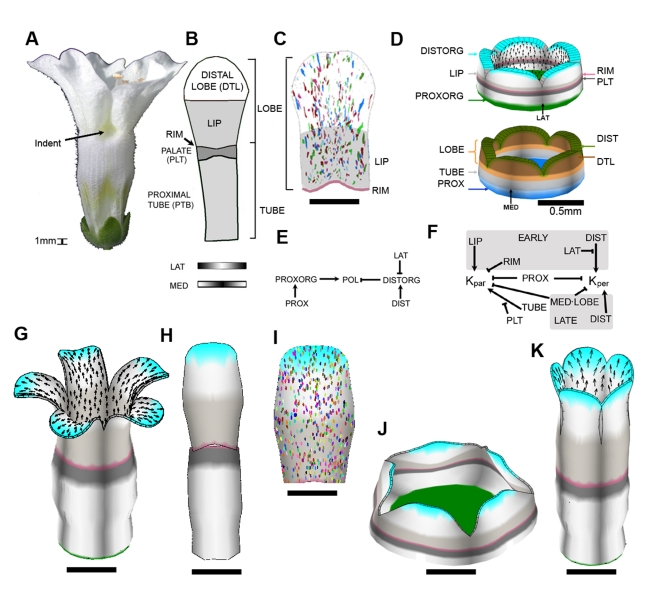
The *cyc dich div* ground state. (A) Mature (day 24) *cyc dich div* flower: all petals are identical and bilaterally symmetrical. (B) Representation of a flattened *cyc dich div* petal, divided into its key regions (see [11]). Patterning along the mediolateral axis of the petal is shown below as distributions of the identity factors LAT (highest at lateral edges) and MED (highest medially). (C) Observed clone pattern on the flattened *div* mutant ventral lobe (induced at ∼ day 14; clones for several lobes overlaid, see Figure S13 for clones induced at other times). (D) Initial canvas (day 10) used for modelling the *cyc dich div* triple mutant corolla. Identity factors are established during the setup phase in domains along the proximodistal and mediolateral axes of each petal. Polarity organisers PROXORG and DISTORG are located proximally and distally. Black arrows indicate gradient of POL. (E) PRN summarising the influences of the polarity organisers PROXORG and DISTORG on polarity through POL (POLARISER). See main text for explanation, and see Model 1 in Text S1B for details of implementation. (F) KRN. Sections shaded grey are active only during the early or late phases (unshaded regions are active throughout early and late phases). See main text for explanation, and see Model 1 in Text S1B for detailed implementation. (G) Shape generated by growing the initial canvas (D) to maturity (day 24). A subset of identity factors and polarity organisers are coloured as in (D). Black arrows indicate gradient of POL. (H) One petal from (G), computationally flattened for comparison with (B). Regions with non-zero Gaussian curvature cannot be flattened without distortion or crushing. We used a heuristic to minimise the distortion [5]. (I) Ellipse pattern on a computationally flattened lobe from (G) (ellipses originated as circles on day 14). Compare with (C). (J) Shape generated by growing the initial canvas (D) to maturity using a variant model with isotropic specified growth (no POL gradient). For detailed implementation, see Model 1, mutant 1 in Text S1B. (K) Shape generated through growing the initial canvas (D) to maturity using a variant model with no inhibition of specified growth rates in medial region of lobes. For detailed implementation, see Model 1, mutant 2 in Text S1B. All scale bars are 5 mm unless otherwise marked.

We first modelled the *cyc dich div* triple mutant corolla: a ground state where all dorsoventral genes are inactive (Figure 4A). The flower is trumpet shaped with slight invaginations at the petal junctions at the rim (arrowed). The key regions in each petal of this ground state have been identified through phenotypic and genetic analysis and comprise the tube (subdivided into proximal tube and palate), rim, and lobe (subdivided into the lip and distal lobe) (Figure 4B, [11]). Each petal also has regional distinctions along the mediolateral axis (indicated by MED and LAT at the bottom of Figure 4B). Clones induced towards the end of the early phase of growth (∼day 14) in ventral petals of *div* mutants tend to yield sectors oriented parallel to the proximodistal axis of the petal (as *CYC* and *DICH* are not expressed ventrally, these are equivalent to petals of the *cyc dich div* mutant). The clones tend to be smaller in the medial region of the lobe and broaden out towards its distal edge (Figure 4C; Figure S13).

For modelling purposes, the initial flower bud was approximated as a cylinder of tissue with five lobes (Figure 4D), similar to the form observed at day 10 (Figure 2). This starting canvas was assigned a pattern of identity factors expressed along the proximodistal and mediolateral axes of its five petal segments during the setup phase. Thus, the corolla was subdivided along the proximodistal axis into TUBE, PLT, RIM, LIP, LOBE, and DTL identities (Figure 4D). The mediolateral axis was defined by LAT and MED, which were given distributions that declined in a graded fashion from the lateral edges and medial region of each petal, respectively (Figure 4D). In accordance with the OPT data, we had two phases over which interactions could vary—an early phase (up to day 14) and a late phase (from day 14 onwards). This was implemented by having EARLY and LATE identity factors that were expressed throughout the canvas.

As maximal growth rate is oriented largely along the proximodistal axis (see clonal analysis above), a simple working hypothesis is that the polarity field is coordinated by two identity factors, one at the base of the corolla, PROXORG, and one at the distal rim, DISTORG (Figure 4D). PROXORG promotes production of the POL signalling factor while DISTORG promotes degradation of POL. The proximodistal gradient in POL is read at each region of the canvas, yielding a local polarity (numerous black arrows in Figure 4D, only shown on the inner surface of the canvas). PROXORG and DISTORG therefore serve to anchor POL distribution, acting as organisers of tissue polarity. As a convention, we show polarity pointing away from PROXORG, which we refer to as a +organiser, and towards DISTORG, which we call a −organiser. PROXORG and DISTORG are activated by the identity factors PROX and DIST, expressed at the base and distal end of the corolla, respectively (see PRN, Figure 4E). DISTORG is also excluded from the lateral edges of each petal by inhibition with LAT.

To capture the overall preferential growth along the proximodistal axis, the basic value of *K_par_* was set to be higher (growth rate of 1.3% per hour) than *K_per_* (0.75% per hour). The rate of growth in thickness *K_nor_* was set at 0.03% per hour, in accordance with OPT measurements. To generate the observed flower shape, regional patterns, and clones of the ground state, specified growth rates were modified through a series of interactions with identity factors, summarised by the KRN in Figure 4F. *K_par_* and *K_per_* were both inhibited by PROX yielding a corolla with a reduced base size. *K_par_* was promoted by TUBE, in the absence of PTL, giving an extended proximal tube. During the early phase *K_par_* was promoted by LIP and inhibited by RIM, yielding petals with a large lip and tight rim. *K_per_* was also promoted by DIST in non-lateral regions to promote broadening of the distal lobe. To capture the observed smaller clone size in the medial regions of the lobe and tendency for clones to broaden distally (Figure 4C), *K_per_* was inhibited by the combination of MED and LOBE (symbolised by MED▪LOBE) and promoted by DIST.

The output of this ground state model was the transformation of the starting canvas to a mature form that was about 500 times larger in area. The overall form resembled the triple mutant corolla, although the invaginations at the petal junctions were not captured (compare Figure 4G with 4A, see also Video S6). Individual petals were also computationally flattened to allow comparison with the experimentally obtained flattened outlines (compare Figure 4H with 4B). Virtual clones were created by marking the canvas with small circles at an early stage and observing the transformed shapes in the mature flower. The resulting ellipse patterns broadly matched the experimentally observed clone patterns in being oriented proximodistally (compare Figure 4I with 4C). The contributions of various aspects of the model to the final shape could also be explored by running variant (mutant) models. A model with the same combinatorial interactions but in which specified growth was isotropic (no POL gradient) led to a squat corolla (Figure 4J). A model with no inhibition of specified growth rates in the medial region of petal lobes led to a corolla with straight rather than bent out lobes (compare Figure 4K with Figure 4G).

### Dorsoventral Genes as Modifiers of Specified Growth Rates

**Figure 5:**
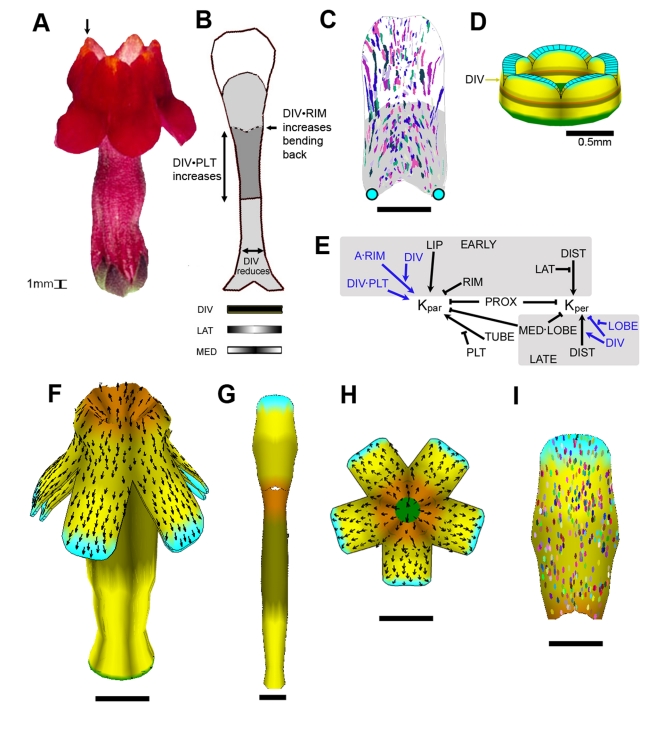
Introducing DIV: The *cyc dich* corolla. (A) Mature (day 24) *cyc dich* flower: all petals are identical, bilaterally symmetrical, and fold back at tube rim. Arrow marks projection at a petal junction. (B) Representation of a flattened *cyc dich* petal, divided into its key regions [11]. Patterning along the mediolateral axis of the petal is shown below as distributions of the identity factors LAT (highest at lateral edges) and MED (highest medially), plus DIV (present everywhere). Compare with Figure 4B. (C) Observed clone pattern on the flattened *cyc dich* lobe (induced at ∼ day 14; clones for several lobes overlaid, see Figure S13 for clones induced at other times). (D) Initial canvas used for modelling the *cyc dich* double mutant corolla. Identity factors are present in domains along the proximodistal and mediolateral axes of each petal. The same identity factors and polarity organisers are present as in Figure 4D, with the addition of DIV (yellow; present everywhere). (E) KRN. Sections shaded grey are active only during the early or late phases (unshaded regions are active throughout early and late phases). Sections coloured blue are influenced by DIV. For detailed implementation, see Model 2 in Text S1B. (F) Shape generated by growing the initial canvas (D) to maturity. A subset of identity factors and polarity organisers are coloured as in (D). (G) One petal from (F) computationally flattened for comparison with (B). (H) Face view of the mature canvas (oblique view shown in (F)). (I) Ellipse pattern on a computationally flattened lobe from (F) (ellipses originated as circles on day 14). Compare with (C). All scale bars are 5 mm unless otherwise marked.

We next incorporated dorsoventral genes in the model, using the working hypothesis that their only role was to modify specified growth rates. The *DIV* gene (which promotes ventral identity) was incorporated first, to capture the *cyc dich* double mutant phenotype. The *cyc dich* mutant has radially symmetrical flowers with an elongated tube and lobes that are strongly bent back at the rim (Figure 5A). The lobes have characteristic projections at the petal junctions (arrows in Figure 5A), giving an undulating rim. Molecular genetic shape analysis has shown that *DIV* has three main effects on the petal—reduction of tube width, promotion of palate length, and bending back at the rim (Figure 5B) [11]. The clones in *cyc dich* lobes tend to be oriented towards the petal junctions at the rim, similar to the pattern observed for wild-type ventral petals (compare Figure 5C with ventral lobe in Figure 3A; see also Figure S13).

We added DIV to the ground state model as an identity factor that was expressed throughout the canvas (yellow colour in Figure 5D). DIV modifies the specified-growth rates in several ways, indicated by the blue sections in the new KRN of Figure 5E. During the early phase, DIV promotes *K_par_* in combination with RIM on the inner or adaxial (*A*) surface of the canvas, leading to bending back of the lobes. This effect of DIV is in accordance with RNA in situ hybridisations, which show enhanced *DIV* and cell cycle gene expression in the adaxial rim region [10],[30]. DIV also promotes *K_par_* in the early phase in combination with PLT, leading to a longer palate. During the late phase, DIV inhibits *K_per_* in the absence of LOBE, leading to reduced petal width, and also enhances the promotion of DIST on *K_per_*, broadening the distal lobe (see Video S7).

The resulting corolla and flattened petal shapes (Figure 5F–I) broadly resemble those for the *cyc dich* double mutant (Figure 5A–C), although the periodic projections at the rim are not captured. A more significant discrepancy between the model and real corollas is that the ellipse patterns in the lobes did not have orientations that diverged outwards towards foci at petal junctions as observed for clones (compare Figure 5I and 5C).

**Figure 6:**
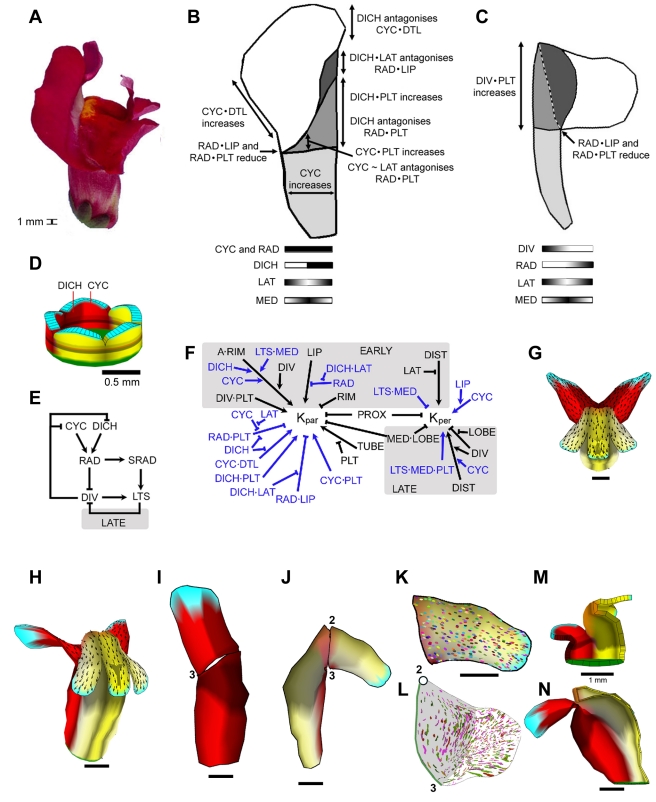
Introducing CYC, DICH, and RAD: The wild-type corolla. (A) Mature (day 24) wild-type flower. (B) Representation of a flattened dorsal petal, divided into its key regions [11]. Patterning along the mediolateral axis of the petal is shown below as distributions of the identity factors LAT (highest at lateral edges), MED (highest medially), DICH (present in the dorsal half), plus CYC and RAD (present everywhere). (C) Representation of a flattened lateral petal, divided into its key regions. Patterning along the mediolateral axis of the petal is shown below as distributions of the identity factors LAT and MED, plus DIV (highest at the most ventral edge) and non-autonomous RAD (highest at the most dorsal edge). (D) Initial canvas used for modelling the wild-type corolla. Identity factors are present in domains along the proximodistal and mediolateral axes of each petal. The same identity factors and polarity organisers are present as in Figure 5D, with the addition of CYC (bright red; present throughout the dorsal petals), RAD (not shown—same distribution as CYC), and DICH (red-brown: present in the dorsal halves of the dorsal petals). (E) Gene Regulatory Network (GRN). Section shaded grey is active only during the late phase. These interactions are supported by genetic data as described in the main text. For detailed implementation, see Model 3 in Text S1B. (F) KRN incorporating CYC, DICH, and RAD (blue sections). CYC promotes *K_per_*, enhanced by LIP, leading to broadening of the dorsal petals. CYC and DICH promote *K_par_* in combination with PLT, leading to increased palate length. CYC promotes *K_par_* in combination with DTL in the absence of DICH leading to enhanced growth of the non-medial region of the dorsal lobe. DICH promotes *K_par_* in combination with PLT leading to extension of the dorsal palate. RAD inhibits *K_par_* in combination with LIP or PLT (in the absence of CYC or DICH modulated by LAT), reducing the length of the lip and palate regions. We also assume that LTS inhibits *K_per_* at early stages, consistent with observations on reduced lateral petal width observed with OPT, and that CYC and DICH modulate relative growth on the two surfaces of the canvas, leading to bending back of the dorsal lobes. For detailed implementation, see Model 3 in Text S1B. (G) Face view of the shape generated by growing the initial canvas (D) to maturity (day 24). (H) Oblique view of the shape generated by growing the initial canvas (D) to maturity (day 24). (I) Dorsal petal from (H) computationally flattened for comparison with (B). Numbers refer to positions as shown in Figure 1C. (J) Lateral petal from (H) computationally flattened for comparison with (C). Numbers refer to positions as shown in Figure 1C. (K) Ellipse pattern on a computationally flattened lateral lobe from (H) (ellipses originated as circles on day 14). (L) Observed clone pattern on the flattened lateral lobe (induced at ∼ day 14; clones for several lobes overlaid). Dot marks point towards which clones tend to be oriented. Compare with (K). (M) Longitudinal section view of canvas at day 14 when ventral petal has arched over (compare with Figure 2). (N) Longitudinal section view of canvas at maturity (day 24). Note that the ventral tube bulges out, in contrast to what is observed experimentally (Figure 2). All scale bars are 5 mm unless otherwise marked.

To capture wild-type development, *CYC*, *DICH*, and *RAD* (three genes known to promote dorsal identity) were next incorporated in the model. Wild-type flowers show marked dorsoventral asymmetry (Figure 6A). The effect of each dorsoventral gene on different petal regions of the mature dorsal and lateral petals has been established through shape analysis of various mutant and over-expression lines (Figure 6B,C) [11]. In accordance with observed expression patterns [6], the CYC identity factor was activated in two adjacent petals of the starting canvas defining the dorsal domain, while DICH was expressed in the most dorsal half of these petals (Figure 6D). Subsequent interactions between the dorsoventral genes were modelled through a GRN based on known gene interactions (Figure 6E). CYC and DICH activate RAD, while RAD inhibits DIV activity [7]. This gives a wild type with CYC, DICH, and RAD in the dorsal petals and DIV activity restricted to the lateral and ventral petals. Since the *rad* mutant is known to have a strongly ventralised phenotype [7], we also assumed that RAD protects CYC and DICH from inhibition by DIV through an inhibitory loop (although other formulations might be possible). The non-autonomous effect of the dorsal identity genes on lateral petals [7] was implemented by RAD activating production of a signalling factor called SRAD that could propagate into the lateral domain. Together with DIV, SRAD activates an identity factor LATERALS (LTS). LTS in turn inhibits DIV at later stages, accounting for the observed later restriction of DIV expression to more ventral regions [10].

*CYC*, *DICH*, and *RAD* have been shown to have several region-specific effects on mature corolla shape (Figure 6B and C) [11]. To account for these effects, we incorporated CYC, DICH, and RAD identity factors into the KRN, such that they influenced *K_par_* and *K_per_* in a way that was likely to create the observed effects on shape and size (blue sections of Figure 6F, see legend for details). With these interactions, the canvas grew to form a flower that resembled wild type in some but not all respects (Figure 6G–N, see also Videos S8, S9). One of the major discrepancies was that the ellipse patterns did not match the pattern of clones observed experimentally. In particular, ellipses near the rim of the lateral petal lobes were oriented along the proximodistal axis rather than parallel to the rim (compare Figure 6K with 6L). Another major problem was that the ventral tube bulged out and did not form an elongated palate. This was because after the ventral tube arched over during early stages (Figure 6M), it continued to arch over during later stages (Figure 6N) rather than straightening out as observed experimentally (Figure 2, day 20). Finally, the dorsal petal lobes became splayed out (Figure 6G) through differential growth of the dorsal palate, rather than remaining together as observed in wild type flowers.

**Figure 7:**
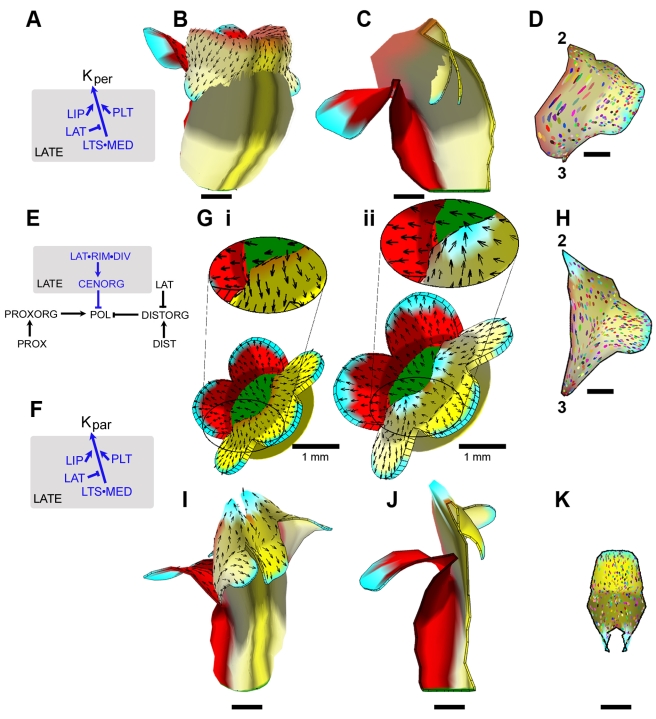
Correction of clonal patterns by modification of the wild-type model. (A) Part of the KRN (rest as in Figure 6F) showing modification of specified growth in the medial region of the lateral petal. For detailed implementation, see Model 4 in Text S1B. (B) Oblique view of mature wild-type canvas grown with the modifications in (A). Black arrows indicate gradient of POL. (C) Longitudinal section (clipped dorsoventrally) of the modified wild-type canvas shown in (B). Note the ventral petal is still bulging outwards. The distal petal lobes grow through the tube as there is no collision detection in the model. (D) Ellipse pattern on a computationally flattened lateral lobe from (B) (ellipses originated as circles on day 14). Compare with Figure 6K and L. Numbers refer to positions shown in Figure 1C. (E) PRN showing addition of a new polarity organiser: CENORG. For detailed implementation, see Model 5 in Text S1B. (F) Part of the KRN (rest as in Figure 6F) showing additional modification in the medial lip and palate of lateral petals. For detailed implementation, see Model 5 in Text S1B. (G) Wild-type canvas before (i) and after (ii) reorientation of growth through activation of CENORG (cyan at the ventral rim indicates location of CENORG). (H) Ellipse pattern on a computationally flattened lateral lobe from the mature canvas grown according to modifications in (E) and (F) (ellipses originated as circles on day 14). Compare with (D) and Figure 6K and L. Numbers refer to positions shown in Figure 1C. (I) Oblique view of mature (day 24) wild-type canvas grown with the modifications described in (E) and (F). (J) Longitudinal section (clipped dorsoventrally) of the modified mature (day 24) wild-type canvas shown in (I). (K) Ellipse pattern on a computationally flattened ventral lobe from the mature canvas grown according to modifications in (E) and (F) (ellipses originated as circles on day 14). All scale bars are 5 mm unless otherwise marked.

To address the discrepancy between modelled and observed clone patterns, we modified the KRN so that the specified growth in the medial region of the lateral and ventral lips was mainly perpendicular rather than parallel to the POL gradient during later stages (Figure 7A). This reduced the arching of the tube (Figure 7B,C) and led to ellipses being elongated parallel to the rim in medial regions of the lateral lobes (Figure 7D). However, the flower did not close, and the lobe shape and ellipse patterns did not capture the observed “flow” pattern of clones. Thus, by assuming that dorsoventral genes influence specified growth rates alone, we were unable to arrive at a simple model that could account for the wild-type shape and growth field. Although our findings do not preclude the possibility that a pattern of specified growth rates might be found that would account for the data, they indicate that such a system would not be straightforward.

### Interactions between Dorsoventral Genes and Polarity Organisers

As an alternative way of addressing the discrepancies between the model and observed shapes and clonal patterns, we explored hypotheses in which the dorsoventral genes influenced organiser activity as well as specified growth rates. Clonal analysis shows that principal orientations of growth tend to become reoriented during the late phase of development towards foci at the ventral petal lobe junctions (Figure 3Biii). We therefore postulated an additional organiser of tissue polarity, CENORG, activated at these foci during the late phase. CENORG was activated by the combination of DIV, PLT, and RIM (see modified PRN, Figure 7E). We assumed that CENORG promoted degradation of POL during the late phase. The resulting POL deficit around the petal junctions reoriented the polarity field (Figure 7G, compare ii with i). Thus, CENORG acts as a −organiser, with polarity arrows being drawn towards it. To account for the elongated clones observed in the medial lip and palate of the lateral petals, we also assumed that identity factors enhance specified growth rates in these regions during the late phase (see modification to KRN in Figure 7F).

The pattern of ellipses generated by the model showed a good match with those observed experimentally. In particular, ellipses in the lip region of the wild-type lateral lobes were oriented parallel to the tube rim, while more distal ellipses were oriented proximodistally, similar to the observed “flow” pattern (compare Figure 7H with Figure 6L). Similarly, ellipses in the ventral petal were oriented towards the petal junctions, as observed with ventral clones (Figure 7K). Most significantly, the introduction of CENORG prevented the ventral tube from bulging outwards and allowed the tube and palate to elongate during the late phase of growth, in agreement with OPT data (Figure 7I,J). Thus, activating CENORG through DIV not only accounts for observed clonal patterns but automatically corrects discrepancies in flower morphogenesis and final shape.

**Figure 8:**
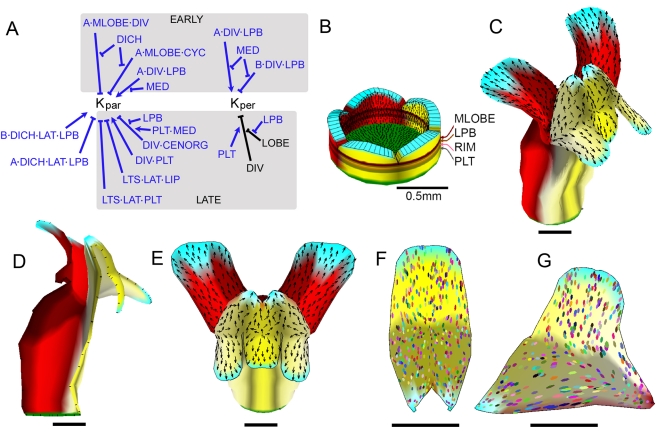
Further modification to the wild-type corolla model. (A) Part of the KRN (rest as in Figures 6F; 7F) showing modifications to specified growth rates that improve the corolla shape. DICH and LAT with LPB promote *K_par_* on the *B* (abaxial) surface and inhibit on the *A* (adaxial) surface leading to the dorsal petal lobes bending forwards. During the early stage, MLOBE in combination with CYC or DIV (but in the absence of DICH) inhibits *K_par_* on the *A* surface, leading to the lobes bending forward. DIV in combination with LPB (but in the absence of MED) promotes *K_par_* and *K_per_* on the *A* surface, while inhibiting *K_per_* on the *B* surface, leading to bulging of the ventral petal junctions. During the late stage, LTS in combination with LAT inhibits *K_par_* in the lip and palate, preventing excessive growth of lateral boundaries. Growth of the ventral palate is modulated by DIV promoting *K_par_* in combination with PLT, while DIV in combination with CENORG inhibits *K_par_* in the absence of LPB and enhanced by PLT and MED, restricting growth of the distal palate. Inhibition of *K_per_* by DIV is promoted by PLT, and by LPB in the LOBE, leading to a narrow ventral palate and lip. For detailed implementation, see Model 6 in Text S1B. (B) Initial (day 10) distribution of additional identity factors used in the model, MLOBE and LPB. (C–E) Mature canvas generated by model based on modifications in (A) shown in oblique view (C), longitudinal section (D), and face view (E). (F–G) Ellipse patterns on computationally flattened ventral (F) and lateral (G) lobes from the mature canvas of the wild-type model incorporating changes in (A) (ellipses originated as circles on day 14). All scale bars are 5 mm unless otherwise indicated.

Building on this model, we introduced further modifications to the KRN so as to generate a flower that resembled wild type more closely (Figure 8A). This involved introducing some further identity factors (LPB and MLOBE, Figure 8B) that modulated specified growth in subregions of the lobe. The resulting flower showed a better match to wild type (Figure 8C–E) and gave ellipse patterns similar to clone patterns observed on ventral and lateral petals (compare Figure 8F,G with Figure 3Ai and Figure 6L).

**Figure 9:**
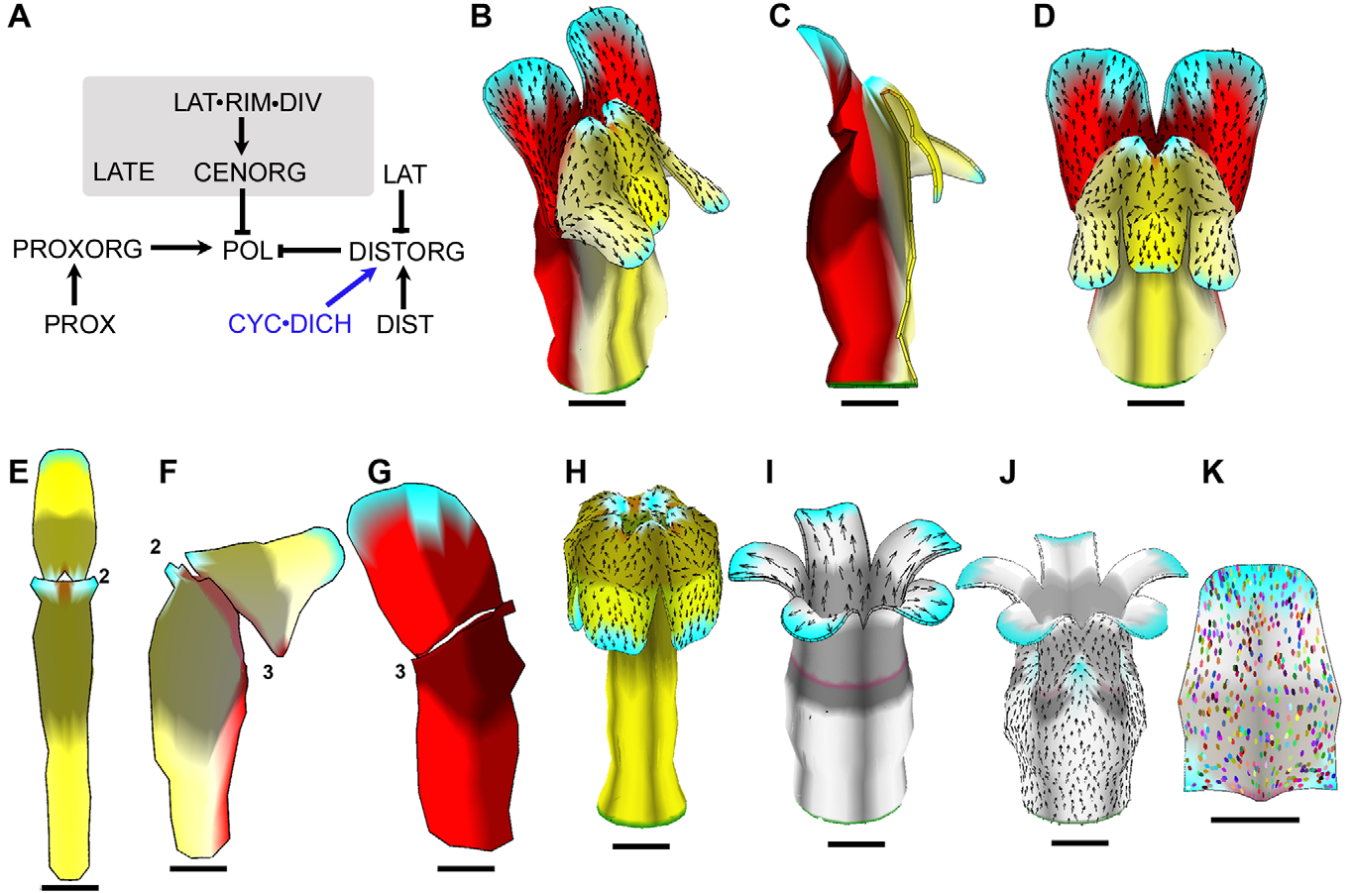
Final version of wild-type corolla model and exploration of mutant forms. (A) Modified PRN incorporating effect of CYC and DICH on DISTORG. For detailed implementation, see Model 7 in Text S1B. (B–D) Mature canvas generated by model incorporating changes in (A), shown in oblique view (B), longitudinal section (C), and face view (D). (E–G) Flattened ventral (E), lateral (F), and dorsal (G) petals from wild-type model shown in (B–D). (H–I) Mature (day 24) canvas from model shown in (B–D), in which CYC and DICH are inactive (H, compare to *cyc dich* mutant Figure 5A), or in which CYC, DICH, and DIV are inactive (I, compare to *cyc dich div* mutant in Figure 4A). (J) Mature (day 24) canvas from a genotype lacking CYC, DICH, and DIV based on a modified model in which CENORG has weak activity in the absence of DIV. Note that there are now invaginations at the petal junctions (cyan areas), similar to those seen in Figure 4A. For detailed implementation, see Model 8 in Text S1B. (K) Ellipse patterns on a computationally flattened petal lobe from (J) (ellipses originated as circles on day 14). All scale bars are 5 mm.

One remaining problem with the model was that the dorsal lobes were much more splayed out (Figure 8E) than observed in wild type, resembling the petals of *dich* mutants [6]. This arises because of the curvature of the tube generated by differential growth of its dorsal regions. To address this issue, we again modulated polarity organiser activity with the dorsoventral genes. We hypothesised that DICH in combination with CYC promotes DISTORG activity (see modified PRN in Figure 9A), as this would be expected to shift specified growth orientations dorsally. This corrected the splaying out of the lobes and also gave dorsal petals with a shape that matched those of wild type more closely (Figure 9B–G).

Thus, by incorporating effects of dorsoventral genes on polarity organisers as well as specified growth rates, we arrived at a model that could account for the main morphogenetic features of the Snapdragon corolla. As a further evaluation of the model, quantitative shape analysis was used to compare multiple genotypes in which particular genes were inactivated or over-expressed in various combinations (*div*, *cyc dich*, *rad*, *backpetals*, *35S::RAD*, *div 35S::RAD*, *cyc dich 35S::RAD*, *rad 35S::RAD*, *backpetals 35S::RAD*). The corolla shapes generated by the model showed a good match with those obtained experimentally [11].

Examination of mutant shapes generated by the final model revealed features not captured by earlier models. For example, unlike our earlier model of the *cyc dich* mutant, the corresponding mutant of the new model produces a shape with periodic projections at the rim, matching experimental observations (compare Figure 9H with Figure 5A,F). The projections arise automatically because of the way specified growth is reoriented towards the petal junctions through activity of CENORG. Projections at petal junctions are not generated in the *cyc dich div* mutant (Figure 9I) as this genotype lacks CENORG activity (CENORG depends on DIV). This is in contrast to experimental observations that reveal slight projections (invaginations) at these positions (Figure 4A). We therefore modified the model such that CENORG is weakly expressed in the absence of DIV. This yields a *cyc dich div* triple mutant with invaginations at the junctions, while maintaining a largely proximodistal orientation of ellipses (Figure 9J,K). These results suggest that DIV boosts CENORG activity rather than being absolutely required for CENORG.

## Discussion

Morphogenesis of the *Antirrhinum* corolla reflects a growth field in which rates and orientations of growth are highly coordinated. Our working hypothesis is that rates are specified by local levels of identity or signalling factors, while orientations depend on underlying tissue polarity, anchored at specific regions called tissue polarity organisers. Polarity information propagates away (+organisers) or towards (−organisers) organisers and is used to define the principal orientations of specified growth within the plane of the canvas. Genes may influence shape by modifying specified growth rates parallel or perpendicular to local polarity and/or by modifying tissue polarity. These genetic effects are mediated by three interconnected regulatory networks: GRN, PRN, and KRN. As more details about the genetic control of growth and polarity become known, it should be possible to integrate these networks further.

Models in which genes modulate specified growth rates along a proximodistal pattern of tissue polarity can account for many aspects of floral morphogenesis but leave certain features unexplained. In particular, such models do not capture the observed pattern of clones and growth orientations on the lateral and ventral petals. They also do not generate the correct flower shape. The ventral tube swells out instead of extending during the later stages of development, the ventral palate fails to elongate, and the dorsal petals splay apart.

The defects in the ventral and lateral petals' growth orientations can be corrected by introducing an additional organiser, CENORG, promoted by DIV at the rim and lateral petal boundaries. As well as capturing growth orientations, this modification has the striking effect of correcting the defect in flower shape, by straightening out the ventral tube and allowing the palate to elongate. This effect on shape reflects the way tissue polarity interacts with the specified growth rates to enhance or reduce tissue curvature [5]. In the case of the *Antirrhinum* flower, proximodistal orientations combined with differential growth lead to arching over of the ventral tube during early stages of development. This effect is then counteracted during later stages by CENORG reorienting polarity, allowing the tube and palate to extend as observed. Introduction of CENORG also accounts for the outgrowths observed at ventral petal junctions in wild type and *cyc dich* mutants. Similar but smaller protrusions are formed at the ventral petal junctions in *div* mutants, suggesting that DIV enhances CENORG rather than being absolutely required for its activity. Thus, the combination of growth analysis and modelling allows the biological significance of the observed clonal patterns to be understood: they reflect the requirement for growth to be reoriented to establish an elongated ventral tube and palate.

Genetic modulation of polarity organisers may also be involved in preventing the dorsal petal lobes from splaying out. A model in which DICH enhances the activity of the distal organiser produces a shape with the dorsal petals held close together. As with CENORG, this effect arises because of the way polarity orientations can counteract curvature induced by differential growth. A model incorporating the effects of DIV and DICH on polarity, as well as specified growth rates, generates a shape and growth field that shows good agreement with experimental data. The model is also supported by quantitative comparisons between predicted shapes and those observed for a range of mutant and over-expression genotypes [11].

### Cellular Basis of Tissue Growth

In principle, specification of growth orientation for a region (e.g., a cell) requires specification of axiality but not necessarily polarity, as local extension may be a bidirectional rather than unidirectional process (axiality can be adequately described with a line, whereas polarity requires a single-headed arrow [5]). In this respect, growth is similar to stress, which has local axiality but no intrinsic polarity. This has led to the suggestion that stresses, rather than molecular signalling mechanisms, may play the key role of organising growth orientations during development [31]. There is also evidence for stresses affecting local growth orientations through changes in the cytoskeleton [21],[32]. However, the morphogenetic possibilities for stress-based systems are constrained by growth orientations being coupled to differential specified growth rates (which lead to stresses). We therefore favour a model based on tissue polarity as this has greater flexibility for generating tissue shapes [5].

Several cellular processes might underlie the propagation of tissue polarity. One is that cells measure the gradient of a diffusing molecule. However, such gradients may be difficult to detect as the tissue grows and gradients become shallower. If there is a limit on the size of gradient that can be read, the pattern of orientations would be disrupted in regions far from the organisers, where the gradient is shallowest. This problem might be circumvented by fixing the polarity after it falls below a threshold value and/or by local cell-cell signalling. Cell-cell polarity propagation systems have been described for animal tissues [27],[28]. In plants, polar distribution of molecules, such as PIN auxin transporters, indicates that cell polarity is also prevalent [33],[34]. Moreover, a site of high auxin concentration has been proposed to act as an organiser of polarity and pattern for the root [35]. However, it is currently unclear how PIN localisation is established or propagated. An attractive hypothesis is that whatever is controlling the orientation of PINs is also controlling the orientations of specified growth. This hypothesis would predict that PIN orientations should converge or diverge at positions where tissue polarity organisers are located. Such PIN polarity patterns are observed at the proximal and distal boundaries of organs [36], consistent with the locations of PROXORG and DISTORG in our model.

**Figure 10:**
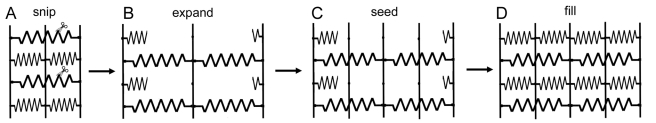
Snip-and-fill mechanism for cell wall growth. Highly schematic model for plant cell wall growth along one axis. (A) Turgor pressure is counterbalanced by load-bearing components in the wall, indicated by stretched springs with cross-links. Loosening of the wall occurs by breaking of covalent or non-covalent bonds. (B) Wall stretches as the load is shifted to the remaining wall components. (C) Space created by expansion allows new cross-links to be inserted, seeding the synthesis or insertion of additional load-bearing components. (D) Following insertion of new material, the original properties of the wall have been restored. The net effect is an increase in the resting length of the wall. Because the insertion of new material in this example is limited by the space created through expansion, residual stresses do not accumulate.

Although stresses may not play the primary role in overall coordination of growth orientations, they nevertheless play an integral part of growth because morphogenesis is a mechanical process [21],[37]. In the case of plants, growth is driven by the continuous expansive turgor pressure within each cell [38]. The resulting stresses are counterbalanced by the cell wall, comprising cellulose microfibrils embedded in a matrix of complex polysaccharides. Breaking or loosening bonds in the wall allows it to stretch in particular orientations, leading to growth [39]. New material is then inserted in the walls to maintain their thickness and strength [23]. A specified growth rate in a particular orientation, as implemented in our model, therefore corresponds to selective weakening or strengthening in particular orientations (determined by cell polarity), followed by insertion of material to maintain wall thickness. Such a “snip-and-fill” mechanism is illustrated in Figure 10. The resultant growth then depends on how the specified growth patterns for various regions of tissue are constrained by mechanical interactions.

If a region of tissue has a specified growth rate different from its neighbours, residual or tissue stresses may be generated [40]–[42]. The extent to which these residual stresses arise and accumulate depends on the mechanism of growth. If new material is inserted into cell walls according to the extent of resultant growth, then the original properties of the wall will be restored and residual stresses will not accumulate. Such a process is captured in our model by relieving residual stresses and strains following the acquisition of new material in the canvas at each growth step. This means that there is effectively feedback between residual stresses and growth. We propose that the previously described feedback from stress orientations to growth [32] relates to this process of stress adjustment, rather than playing a primary role of specifying growth orientations during morphogenesis. It is also possible that residual stresses may accumulate during some stages of development. For example, the mature *Antirrhinum* corolla is held tightly shut by expansive forces in the ventral tube that are balanced by the apposing dorsal corolla. These residual stresses can be revealed by cutting the mature corolla and observing it spring into a new shape. Such forces may arise during the later stages of growth and could be modelled by allowing residual stresses to accumulate to a specified degree.

### Evolution of Shape

Dorsoventral flower asymmetry is a common feature of the Lamiales (the Order to which *Antirrhinum* belongs). However, the formation of flowers that have a closed mouth with a hinged palate (known as the personate form) is restricted to a small but diverse clade within the Lamiales [43]. The *Antirrhinum* corolla model indicates that a key step in the evolution of personate flowers may have been bringing tissue polarity organisers under the control of genes like *DIV* and *DICH*. In particular, the formation of a hinged lower palate matching the upper corolla depends on promotion of CENORG by DIV. It is possible that equivalent morphogenetic changes could have been brought about through changes in patterns of specified growth rates rather than tissue polarity. However, modulating tissue polarity may provide a simpler developmental mechanism for some coordinated changes in form and may therefore have been favoured during evolution. Other evolutionary innovations, such as formation of flower spurs [44], may also involve genes influencing organisers of tissue polarity. Thus, changes in polarity as well as specified growth rates may play a key part in the evolution of complex morphologies.

## Materials and Methods

### Plant Material

*Antirrhinum majus* wild type (JIC Stock 7) and *div-13*, *cyc-608 dich-718*, and *cyc-608 dich^G^ div-80* mutants were grown in the greenhouse at the John Innes Centre. For clonal analysis, plants carrying a transposon at *PALLIDA* were grown at 25°C in constant light [13]. Excision of the transposon was induced by moving the plants to 15°C for 24 h. The developmental stage of a bud at the time of induction was determined by matching bud size and morphology with a standard time course [12].

### 3-D Imaging and Analysis

Flower buds with sepals removed were collected at various developmental stages and stored in 100% ethanol. Buds were prepared for OPT as described previously [17]. Scanning and reconstructions were performed using a Bioptonics OPT Scanner 3001 or a prototype OPT Scanner at the John Innes Centre. 3-D images were analysed using UFEEL software [45], with a 3-D screen and SensAble PHANToM Omni haptic device. UFEEL was also used to measure distances, including lengths and thicknesses of the tube and lobe.

### Clonal Analysis

To record clone patterns, petal parts (Figure 1C) were separated and flattened between microscope slides. Their adaxial surfaces were then imaged using a Kodak DCS Pro 14N camera with a Nikon Nikkor AF 60 f/2.8D lens. Images for each petal part and induction stage were warped to the appropriate mean shape. Clone patterns were segmented and edited using Sector Analysis Toolbox software (available on demand), which was based on principles outlined in [20]. This software also allowed growth parameters to be extracted for each region (for further details, see Text S1A).

## Supporting Information

Figure S1 **Clonal patterns and growth data for wild-type shaped petals, relates to** **Figure 3** **.** (A–E) Clones on petals of several flowers induced at a range of stages: (A) 300 h (day 12.5), (B) 340 h (day 14), (C) 380 h (day 16), (D) 420 h (day 17.5), and (E) 460 h (day 19), warped to a mean petal shape and overlaid, with a different colour used for clones from each petal (see Figure 3A). Two versions (i and ii) are shown at each stage, made by overlaying separate sets of clone images. (F–I) Principal directions of growth for periods: (F) 300–340 h (day 12.5–14), (G) 340–380 h (day 14–16), (H) 380–420 h (day 16–17.5), and (I) 420–460 h (day 17.5–19), shown as short lines scaled according to the value of *K_max_* within each period (see Figure 3B). (J–M) Maximal growth rates (*K_max_*) calculated for periods: (J) 300–340 h (day 12.5–14), (K) 340–380 h (day 14–16), (L) 380–420 h (day 16–17.5), and (M) 420–460 h (day 17.5–19). Scale above (J) is used for (J–M).
(10.01 MB TIF)

Figure S2 **Higher resolution version of Figure S1A (clones at 300 h/day 12.5).**
(9.03 MB TIF)

Figure S3 **Higher resolution version of Figure S1A (clones at 300 h/day 12.5).**
(9.02 MB TIF)

Figure S4 **Higher resolution version of Figure S1B (clones at 340 h/day 14).**
(8.99 MB TIF)

Figure S5 **Higher resolution version of Figure S1B (clones at 340 h/day 14).**
(9.03 MB TIF)

Figure S6 **Higher resolution version of Figure S1C (clones at 380 h/day 16).**
(9.07 MB TIF)

Figure S7 **Higher resolution version of Figure S1C (clones at 380 h/day 16).**
(9.15 MB TIF)

Figure S8 **Higher resolution version of Figure S1D (clones at 420 h/day 17.5).**
(9.04 MB TIF)

Figure S9 **Higher resolution version of Figure S1D (clones at 420 h/day 17.5).**
(9.07 MB TIF)

Figure S10 **Higher resolution version of Figure S1E (clones at 460 h/day 19).**
(9.03 MB TIF)

Figure S11 **Higher resolution version of Figure S1E (clones at 460 h/day 19).**
(9.07 MB TIF)

Figure S12 **Grids used for clonal analysis of wild-type petals, relates to** **Figure 3** **and Tables S1,S2.** (A–D) Grids used for the period 300–340 h: (A) Dorsal lobe, (B) Lateral lobe, (C) Ventral lobe, and (D) Half tube. (E–H) Grids used for the period 340–380 h: (E) Dorsal lobe, (F) Lateral lobe, (G) Ventral lobe, and (H) Half tube. (I–L) Grids used for the period 380–420 h: (I) Dorsal lobe, (J) Lateral lobe, (K) Ventral lobe, and (L) Half tube. (M–P) Grids used for the period 420–460 h: (M) Dorsal lobe, (N) Lateral lobe, (O) Ventral lobe, and (P) Half tube.
(4.82 MB TIF)

Figure S13 **Clonal patterns for dorsoventral mutant petals, relates to** **Figures 4** **and** **5** **.** (A–E) Clones on lobes from several *cyc dich* double mutant flowers induced at a range of stages: (A) 300 h (day 12.5), (B) 330 h (day 14), (C) 350 h (day 15), (D) 380 h (day 16), and (E) 400 h (day 17) (note that developmental timing does not correlate perfectly with wild type), warped to a mean lobe shape and overlaid, with a different colour used for clones from each petal. (F–J) Clones on ventral lobes (in which *cyc* and *dich* are inactive) from several *div* mutant flowers induced at a range of stages: (F) 300 h (day 12.5), (G) 340 h (day 14), (H) 380 h (day 16), (I) 420 h (day 17.5), and (J) 440 h (day 18) (note that developmental timing does not correlate perfectly with wild type), warped to a mean lobe shape and overlaid, with a different colour used for clones from each petal.
(2.85 MB TIF)

Table S1 **Average sector ellipses by region (relates to** **Figure 3** **).** An Excel table showing the lengths of the major axis (*E_maj_*) and minor axis (*E_min_*) and the orientation (*θ*) for the average sector ellipse for each region (see Figure S12; *θ* is given in radians relative to the *x*-axis) for each stage (300 h, 340 h, 380 h, 420 h, and 460 h). There are two sets of data for the middle stages (340 h, 380 h, and 420 h), corresponding to the optimal grids for the two relevant growth steps. The sheets “Dorsal,” “Lateral,” “”Ventral,” and “Tube” each give the average sector ellipse information for the named petal section.
(0.14 MB XLS)

Table S2 **Growth data for each region (relates to** **Figure 3** **).** An Excel table showing the growth along the principal direction of growth (*K_max_*), the growth perpendicular to that within the plane of the petal surface (*K_min_*), the anisotropy (*K_max_*/*K_min_*), and the orientation (*θ*) of the principal direction of growth for each region (see Figure S12) for each step (300–340 h, 340–380 h, 380–420 h, and 420–460 h). The sheets “Dorsal,” “Lateral,” “Ventral,” and “Tube” each give the growth data for the named petal section.
(0.13 MB XLS)

Text S1 (A) Data on clonal analysis. (B) Snapdragon model.
(0.09 MB PDF)

Video S1 **OPT image of flower bud at day 10 (relates to** **Figure 2** **).**
(0.68 MB MOV)

Video S2 **OPT image of flower bud at day 12 (relates to** **Figure 2** **).**
(1.03 MB MOV)

Video S3 **OPT image of flower bud at day 14 (relates to** **Figure 2** **).**
(1.18 MB MOV)

Video S4 **OPT image of flower bud at day 17 (relates to** **Figure 2** **).**
(1.78 MB MOV)

Video S5 **OPT image of flower bud at day 20 (relates to** **Figure 2** **).**
(1.28 MB MOV)

Video S6 **Growth model of the** ***cyc dich div*** **triple mutant (scale constant throughout).** LIP in light grey, PLT in dark grey, −organisers in cyan, +organiser in green. Coloured circles (virtual clones) induced at about 14 d (relates to Figure 4).
(1.31 MB MOV)

Video S7 **Growth model of the** ***cyc dich*** **double mutant (scale constant throughout).** LIP in light grey, PLT in dark grey, DIV in yellow, −organisers in cyan, +organiser in green. Coloured circles (virtual clones) induced at about 14 d (relates to Figure 5).
(1.05 MB MOV)

Video S8 **First growth model of wild-type corolla (scale constant throughout).** LIP in light grey, PLT in dark grey, DIV in yellow, CYC in red, DICH in brown, −organisers in cyan, +organiser in green. Coloured circles (virtual clones) induced at about 14 d (relates to Figure 6 and to Model 3 in Text S1B).
(1.11 MB MOV)

Video S9 **Final growth model of wild-type corolla (scale constant throughout).** Factors shown as in Video S8. Coloured circles (virtual clones) induced at about 14 d (relates to Figure 9 and to Model 7 in Text S1B).
(1.33 MB MOV)

## Abbreviations

ω: rotation rate for a region
*θ*: the principal orientation of growth for a region
*A* surface: one surface of the canvas
*B* surface: the other surface of the canvas
GPT-framework: Growing Polarised Tissue framework
GRN: Gene Regulatory Network
*K_max_*: growth rate along the principal orientation of growth for a region
*K_min_*: growth rate perpendicular to the principal orientation of growth for a region
*K_nor_*: specified growth rate in tissue thickness for a region
*K_par_*: specified growth rate for a region, in the plane of the canvas, parallel to the local polarity
*K_per_*: specified growth rate for a region, in the plane of the canvas, perpendicular to the local polarity
KRN: growth rate (*K*) Regulatory Network
LTS: LATERALS (an identity factor in the corolla model)
OPT: Optical Projection Tomography
+organiser: a region of the canvas that organises polarity with arrows pointing away from it
−organiser: a region of the canvas that organises polarity with arrows pointing towards it
POL: POLARISER (a signalling factor in the GPT-framework that is used to implement polarity propagation—the gradient of POL defines local polarity)
PRN: Polarity Regulatory Network
