# Supplementary figures and images for: Genetic Control of Organ Shape and Tissue Polarity
Source: PLoS Biol. 2010 Nov 9;8(11):e1000537. doi: 10.1371/journal.pbio.1000537 (PMC2976718; doi:10.1371/journal.pbio.1000537)

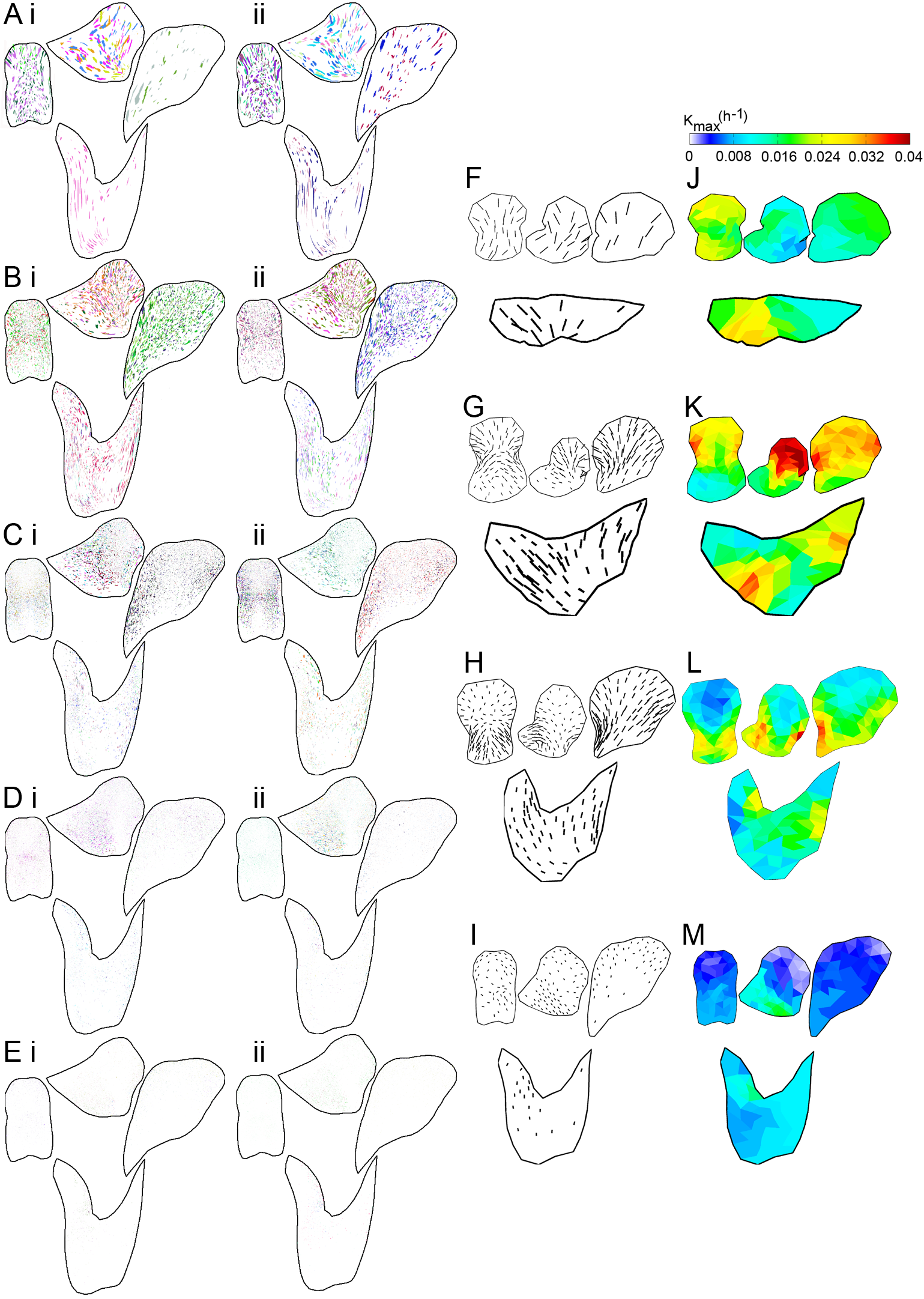

Supplement: Figure S1 — Clonal patterns and growth data for wild-type shaped petals, relates to Figure 3 . (A–E) Clones on petals of several flowers induced at a range of stages: (A) 300 h (day 12.5), (B) 340 h (day 14), (C) 380 h (day 16), (D) 420 h (day 17.5), and (E) 460 h (day 19), warped to a mean petal shape and overlaid, with a different colour used for clones from each petal (see Figure 3A). Two versions (i and ii) are shown at each stage, made by overlaying separate sets of clone images. (F–I) Principal directions of growth for periods: (F) 300–340 h (day 12.5–14), (G) 340–380 h (day 14–16), (H) 380–420 h (day 16–17.5), and (I) 420–460 h (day 17.5–19), shown as short lines scaled according to the value of Kmax within each period (see Figure 3B). (J–M) Maximal growth rates (Kmax) calculated for periods: (J) 300–340 h (day 12.5–14), (K) 340–380 h (day 14–16), (L) 380–420 h (day 16–17.5), and (M) 420–460 h (day 17.5–19). Scale above (J) is used for (J–M). (10.01 MB TIF) [file pbio.1000537.s001.tif]

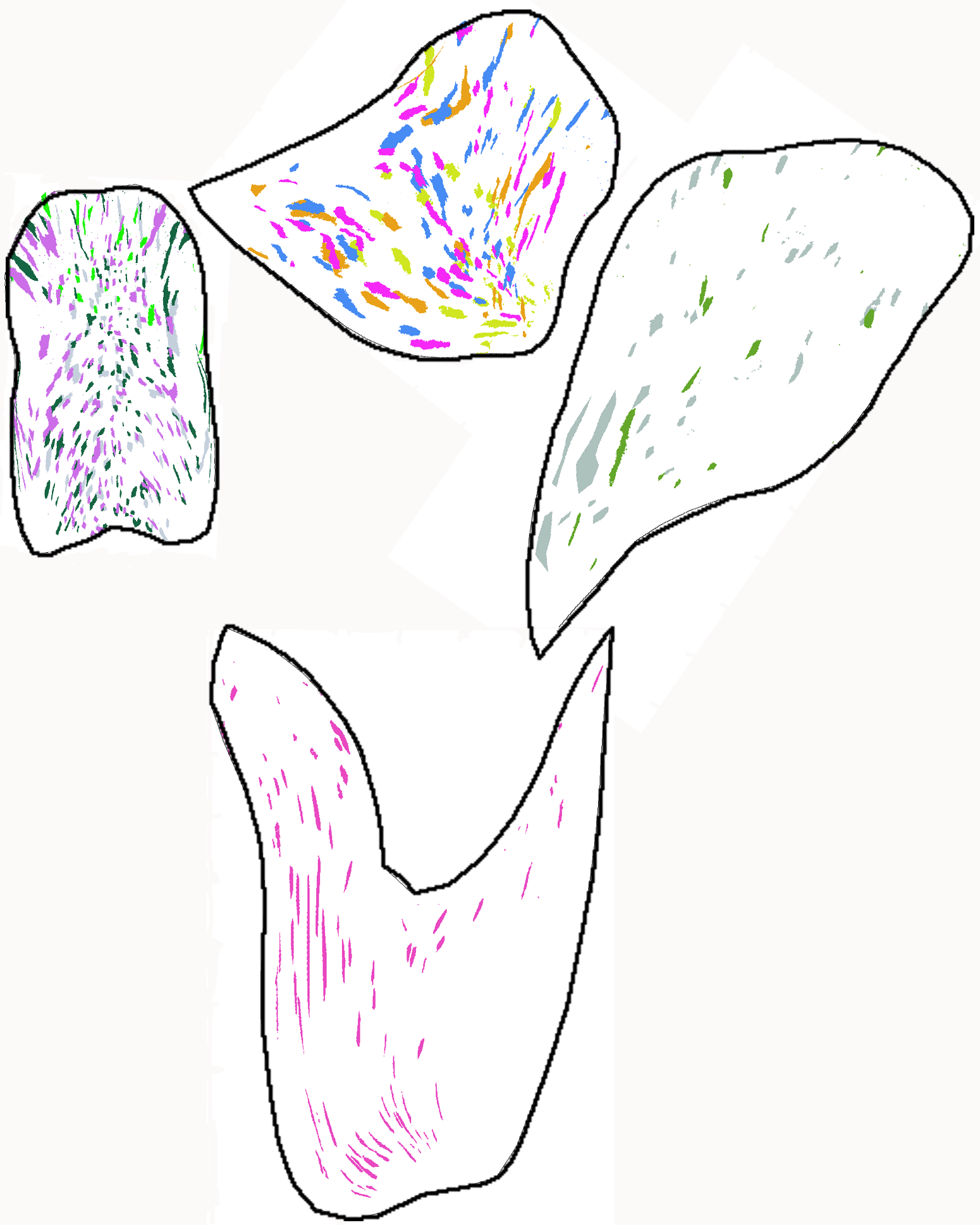

Supplement: Figure S2 — Higher resolution version of Figure S1A (clones at 300 h/day 12.5). (9.03 MB TIF) [file pbio.1000537.s002.tif]

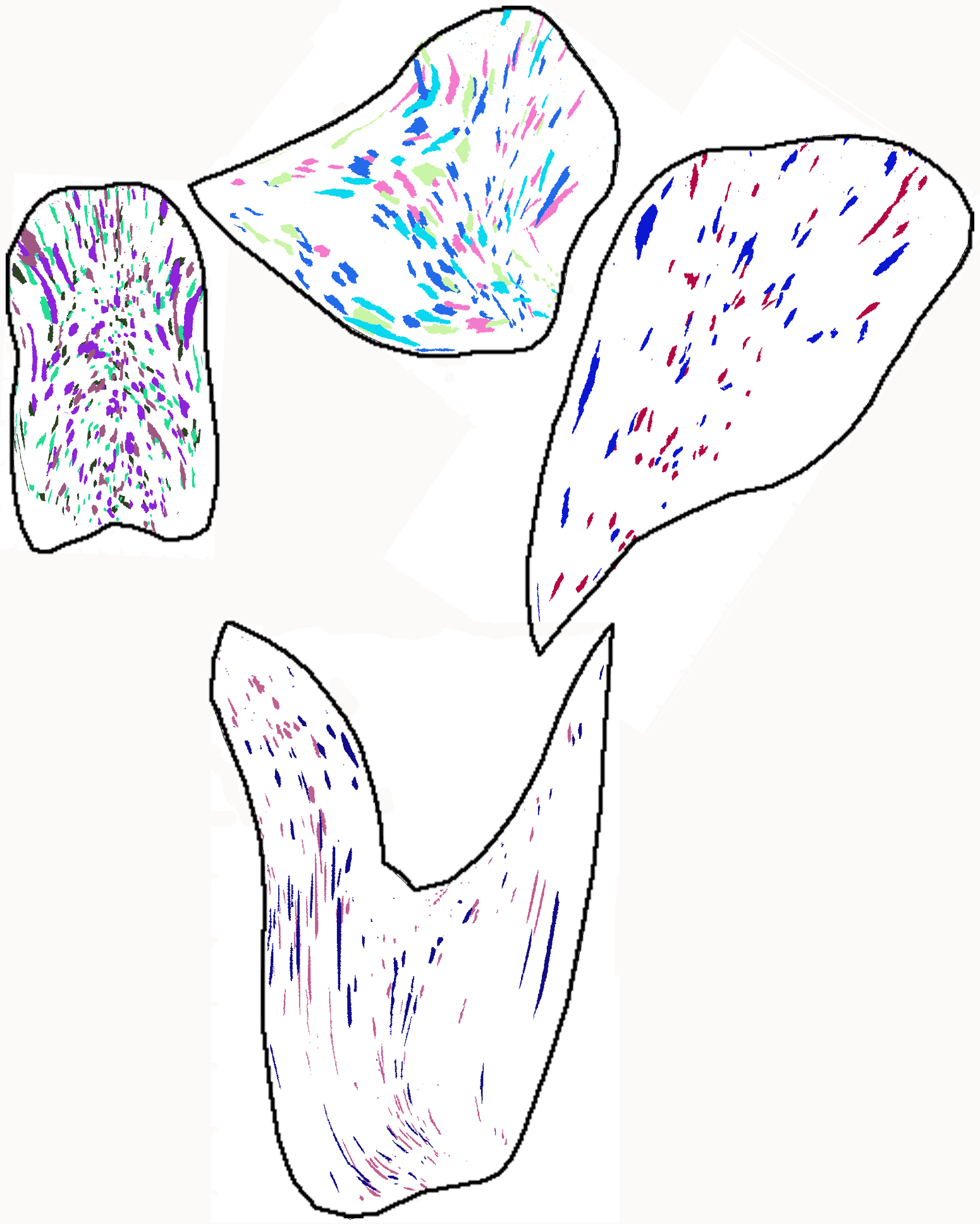

Supplement: Figure S3 — Higher resolution version of Figure S1A (clones at 300 h/day 12.5). (9.02 MB TIF) [file pbio.1000537.s003.tif]

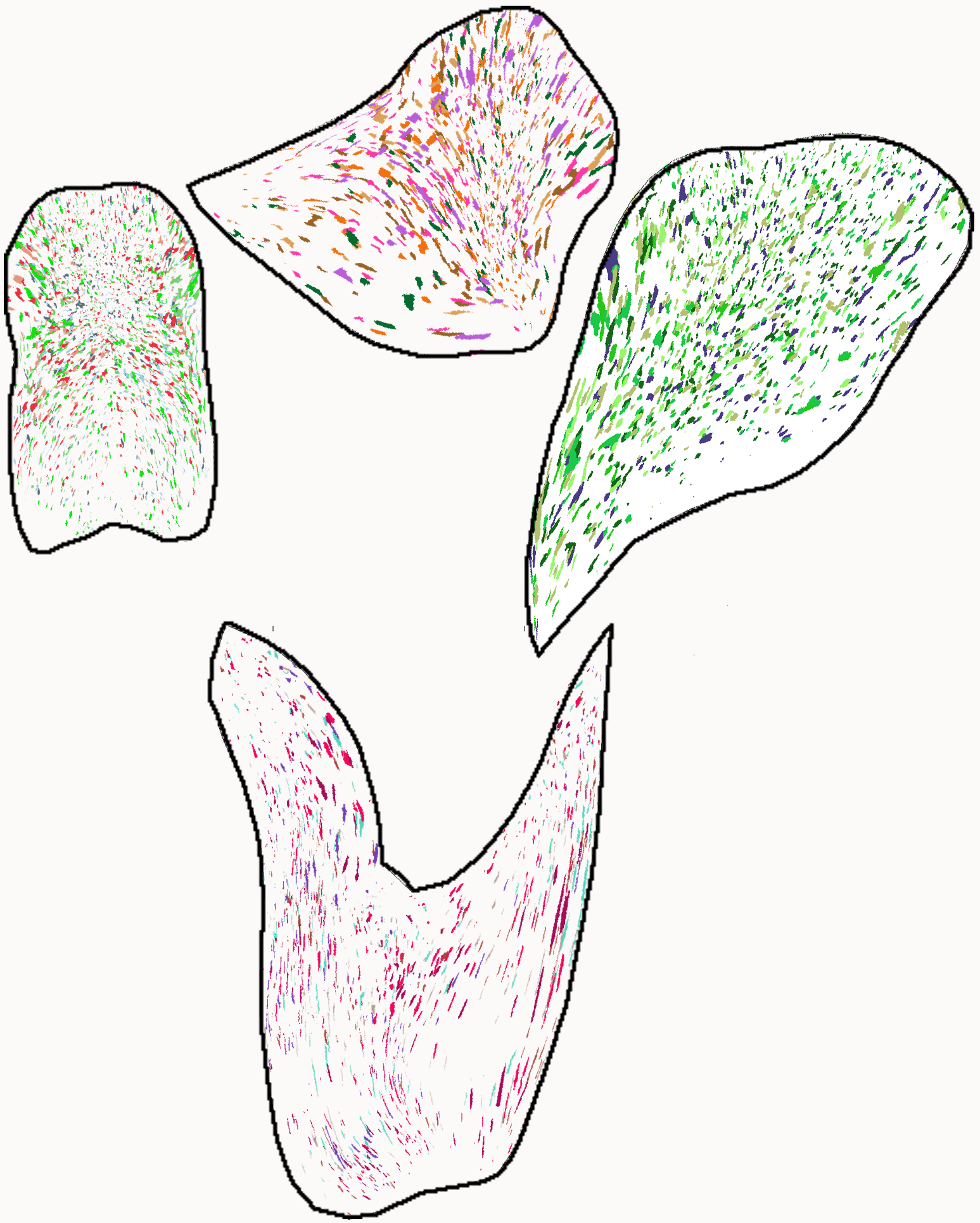

Supplement: Figure S4 — Higher resolution version of Figure S1B (clones at 340 h/day 14). (8.99 MB TIF) [file pbio.1000537.s004.tif]

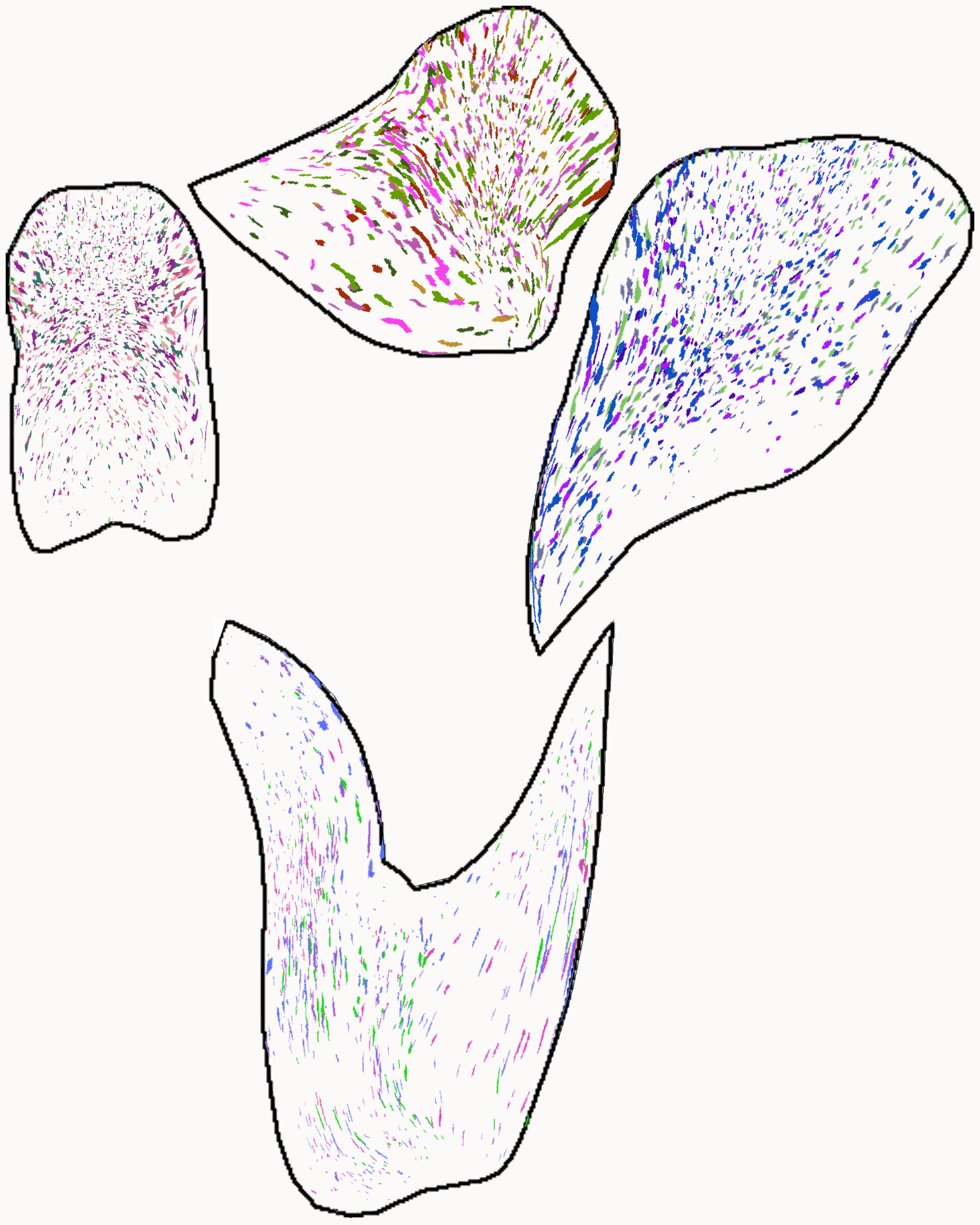

Supplement: Figure S5 — Higher resolution version of Figure S1B (clones at 340 h/day 14). (9.03 MB TIF) [file pbio.1000537.s005.tif]

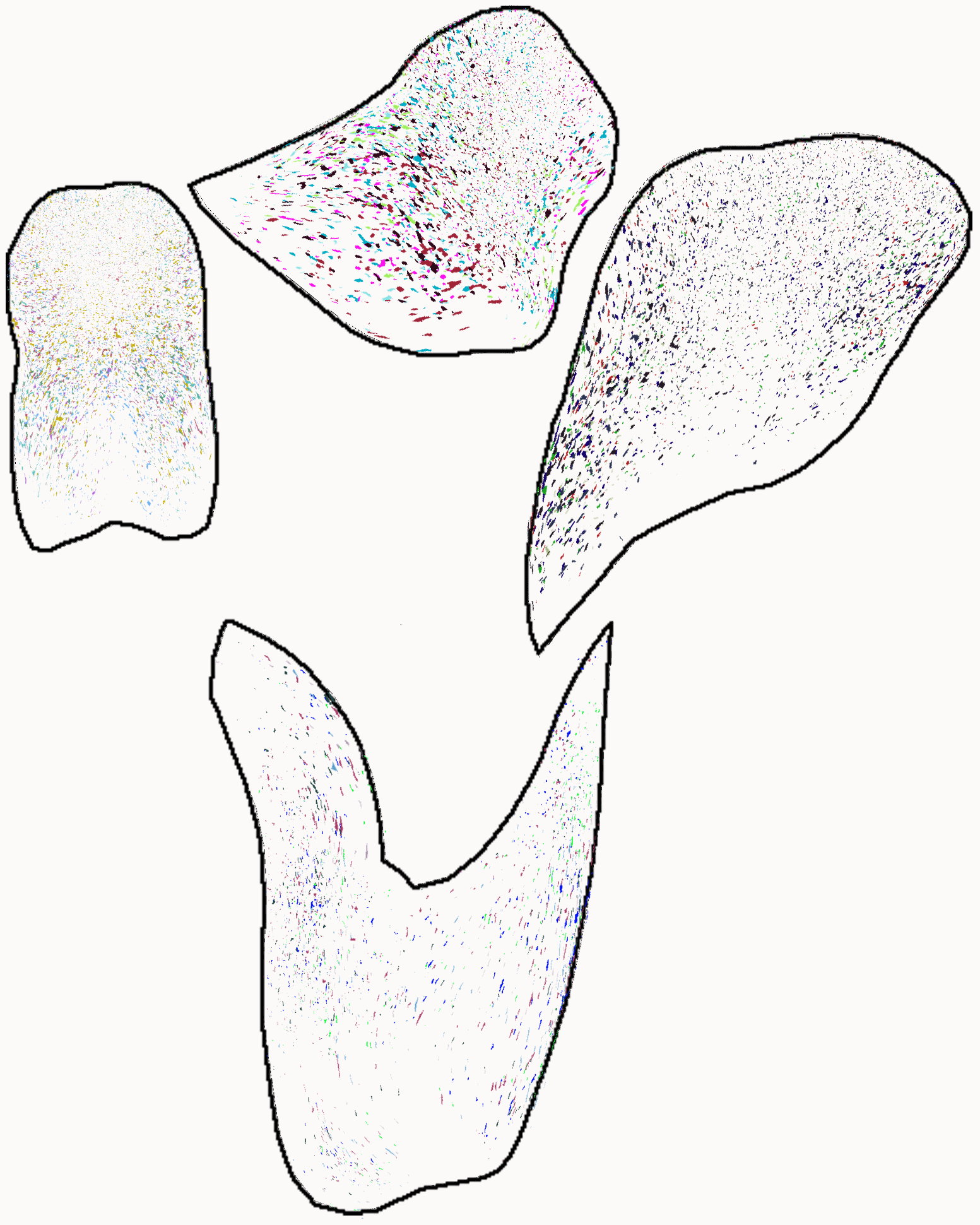

Supplement: Figure S6 — Higher resolution version of Figure S1C (clones at 380 h/day 16). (9.07 MB TIF) [file pbio.1000537.s006.tif]

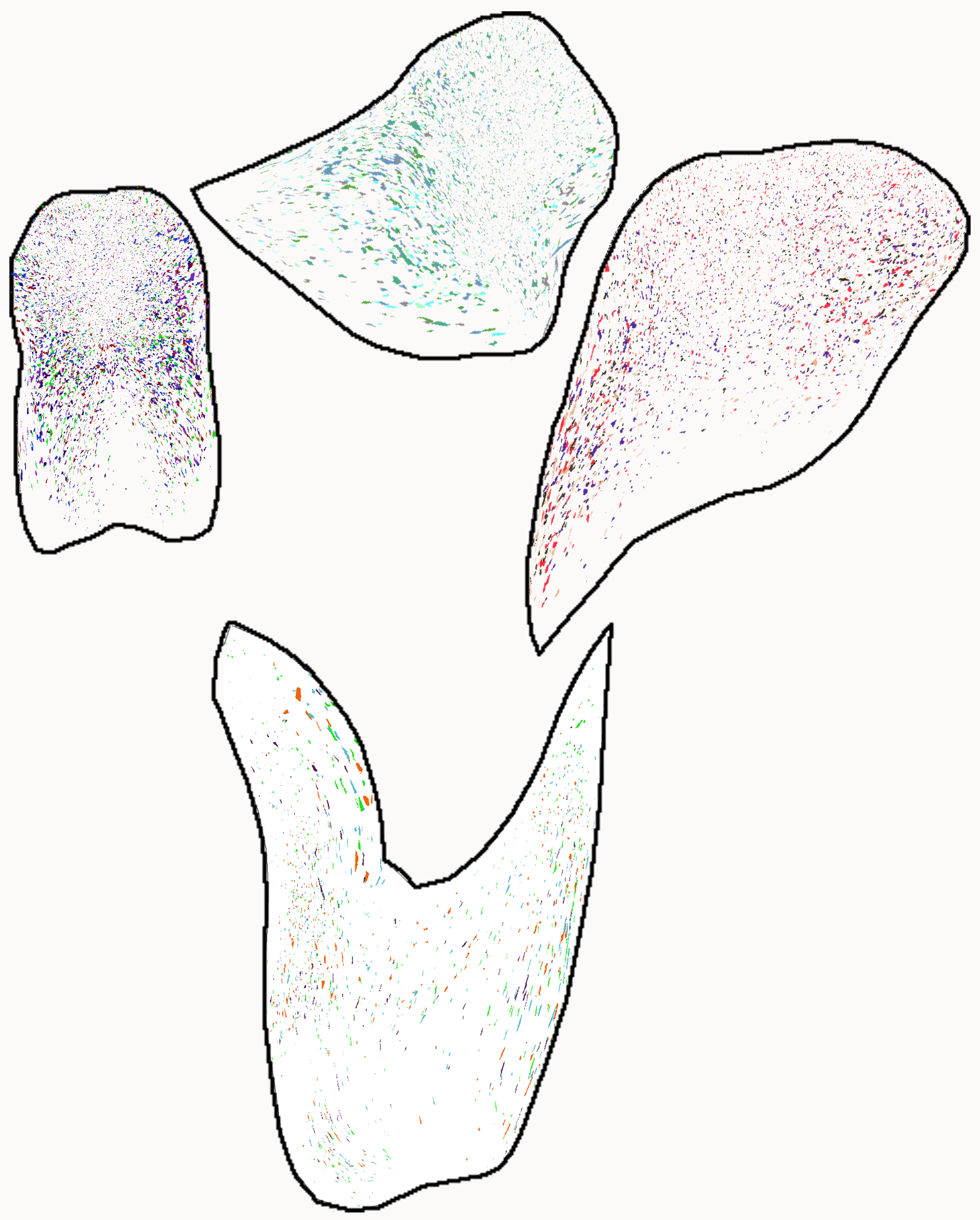

Supplement: Figure S7 — Higher resolution version of Figure S1C (clones at 380 h/day 16). (9.15 MB TIF) [file pbio.1000537.s007.tif]

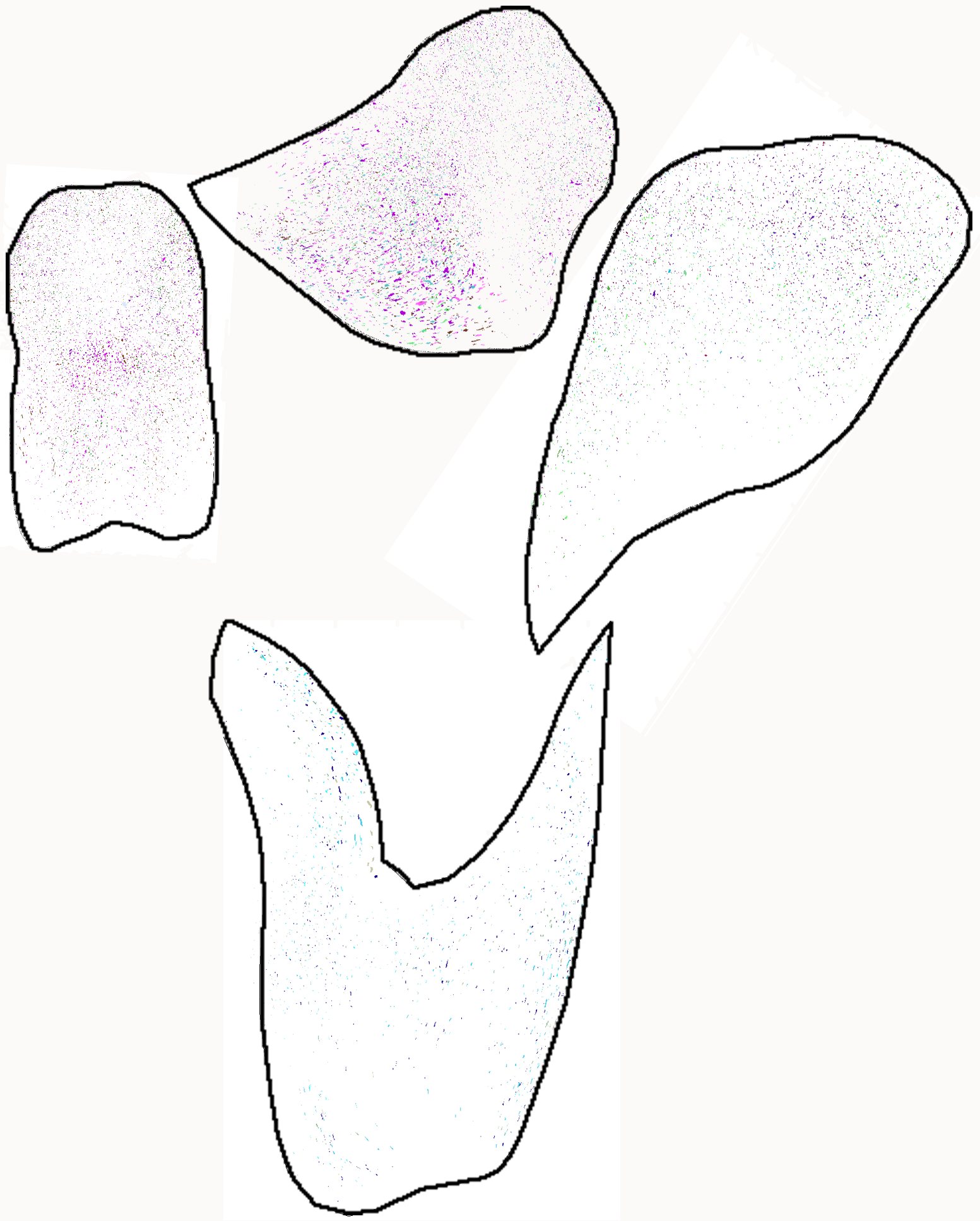

Supplement: Figure S8 — Higher resolution version of Figure S1D (clones at 420 h/day 17.5). (9.04 MB TIF) [file pbio.1000537.s008.tif]

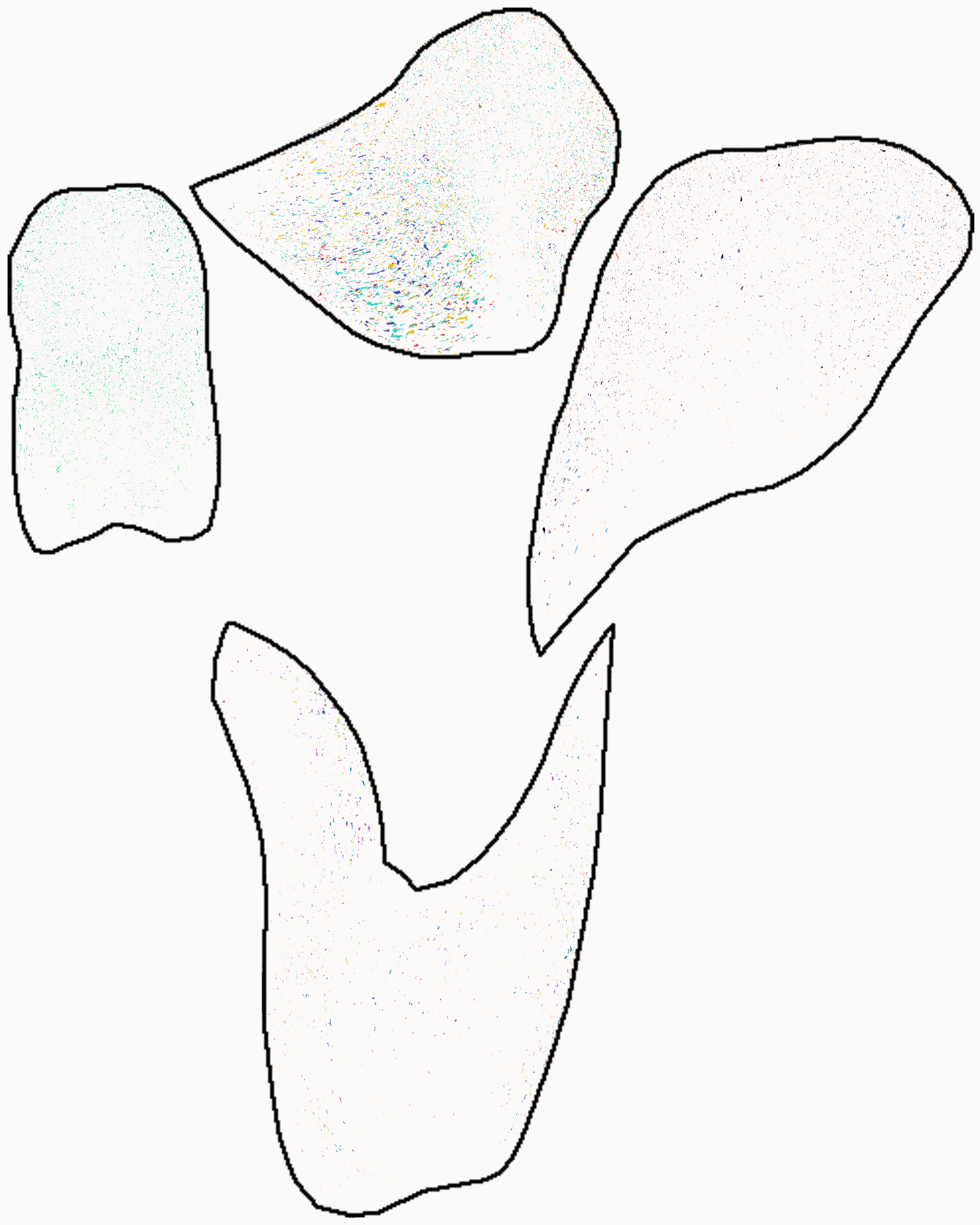

Supplement: Figure S9 — Higher resolution version of Figure S1D (clones at 420 h/day 17.5). (9.07 MB TIF) [file pbio.1000537.s009.tif]

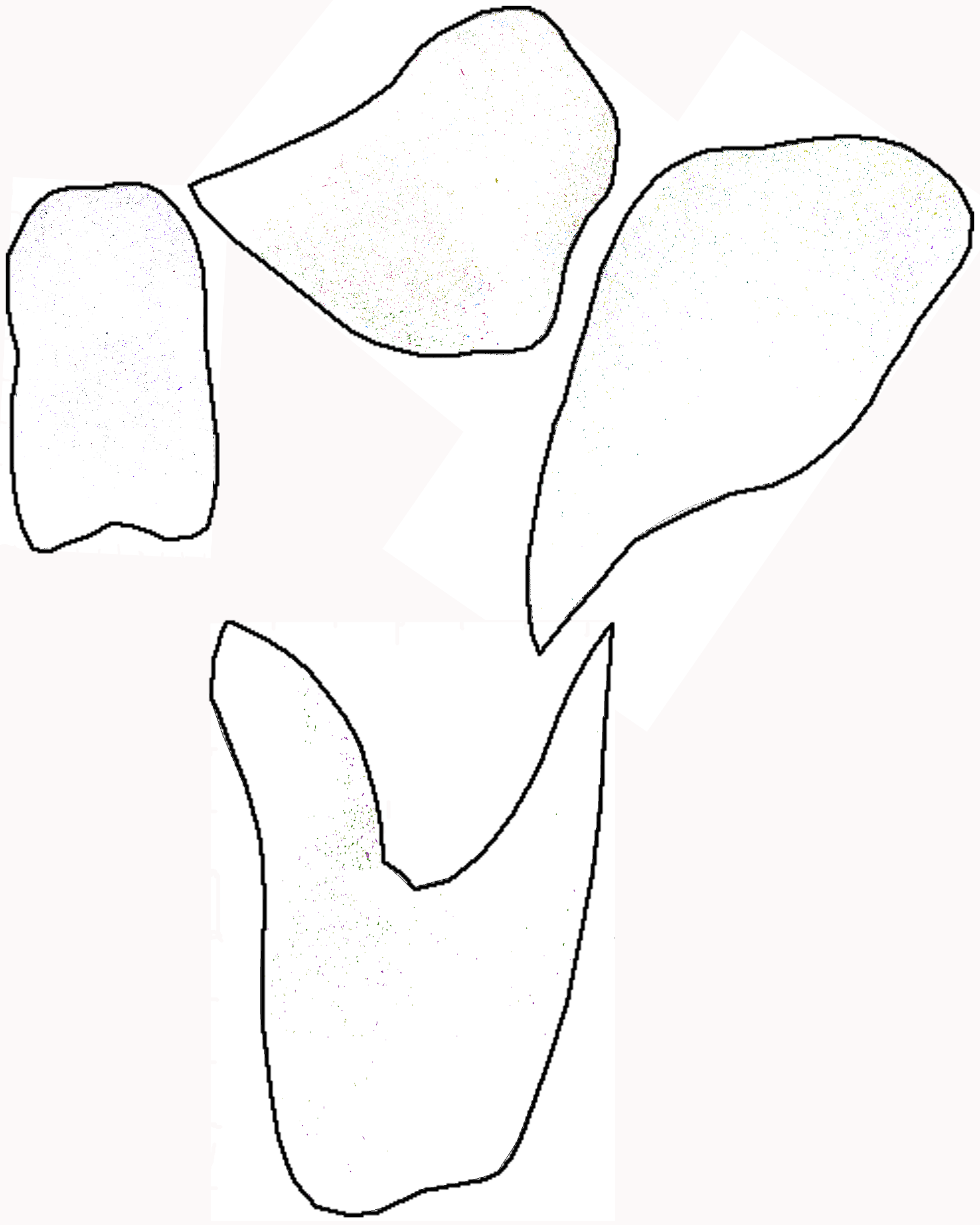

Supplement: Figure S10 — Higher resolution version of Figure S1E (clones at 460 h/day 19). (9.03 MB TIF) [file pbio.1000537.s010.tif]

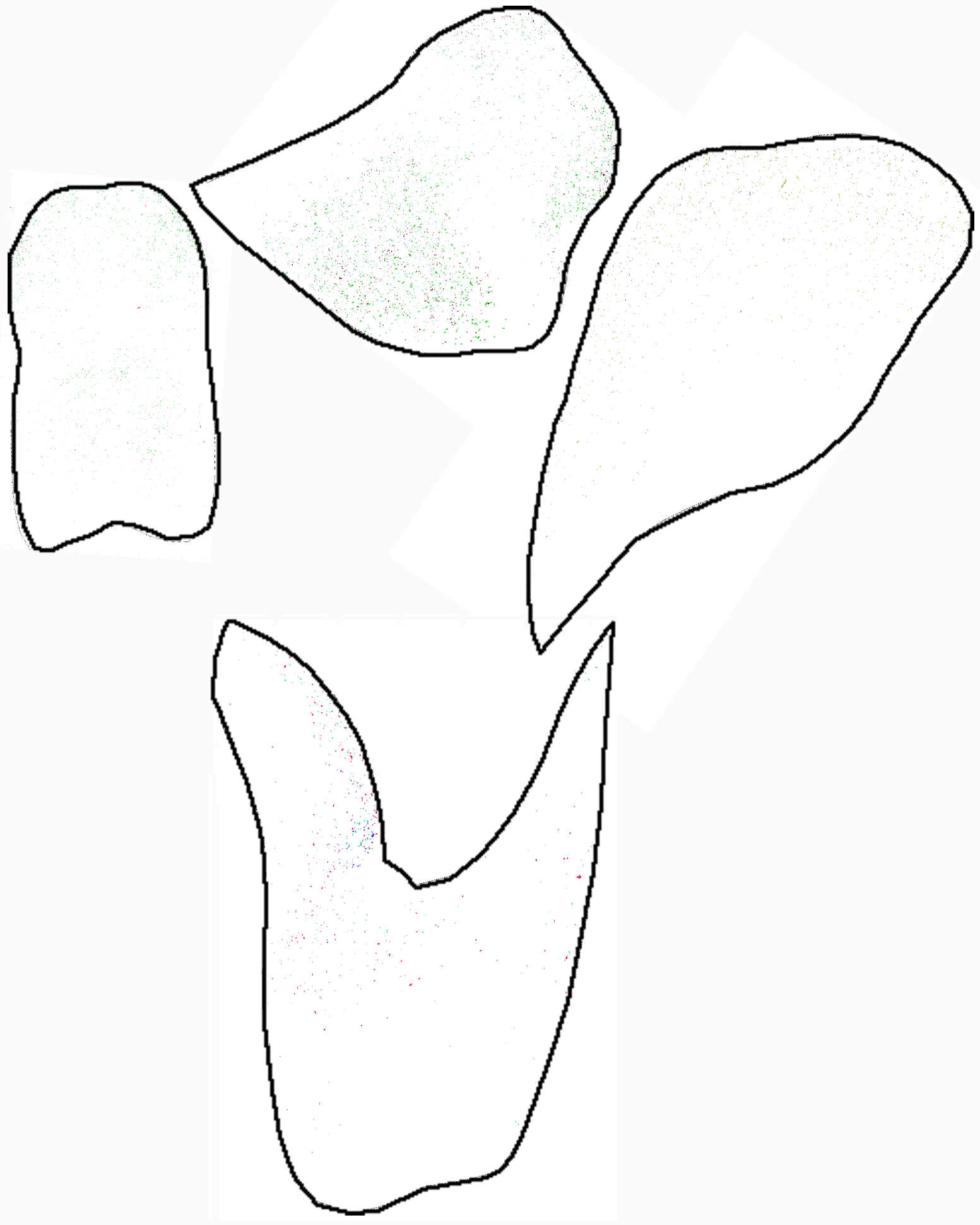

Supplement: Figure S11 — Higher resolution version of Figure S1E (clones at 460 h/day 19). (9.07 MB TIF) [file pbio.1000537.s011.tif]

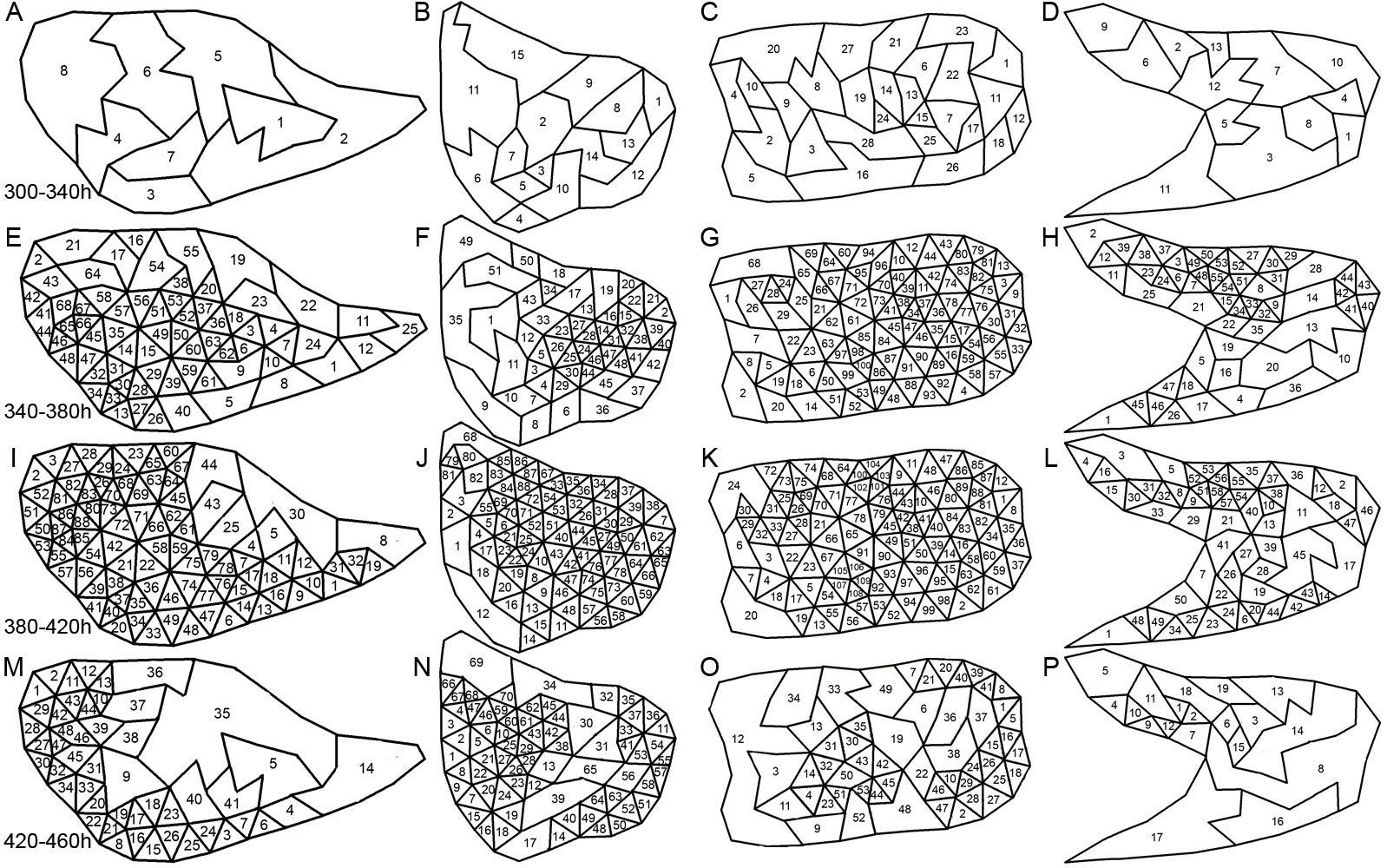

Supplement: Figure S12 — Grids used for clonal analysis of wild-type petals, relates to Figure 3 and Tables S1,S2. (A–D) Grids used for the period 300–340 h: (A) Dorsal lobe, (B) Lateral lobe, (C) Ventral lobe, and (D) Half tube. (E–H) Grids used for the period 340–380 h: (E) Dorsal lobe, (F) Lateral lobe, (G) Ventral lobe, and (H) Half tube. (I–L) Grids used for the period 380–420 h: (I) Dorsal lobe, (J) Lateral lobe, (K) Ventral lobe, and (L) Half tube. (M–P) Grids used for the period 420–460 h: (M) Dorsal lobe, (N) Lateral lobe, (O) Ventral lobe, and (P) Half tube. (4.82 MB TIF) [file pbio.1000537.s012.tif]

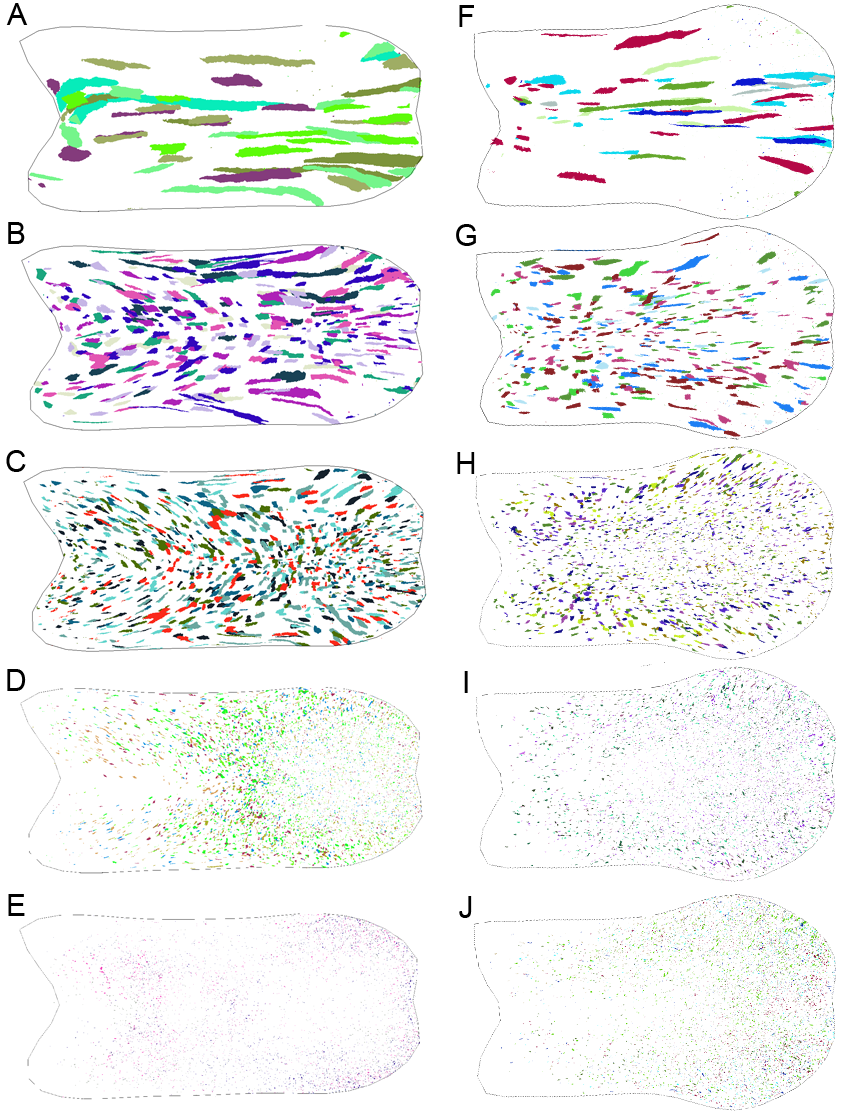

Supplement: Figure S13 — Clonal patterns for dorsoventral mutant petals, relates to Figures 4 and 5 . (A–E) Clones on lobes from several cyc dich double mutant flowers induced at a range of stages: (A) 300 h (day 12.5), (B) 330 h (day 14), (C) 350 h (day 15), (D) 380 h (day 16), and (E) 400 h (day 17) (note that developmental timing does not correlate perfectly with wild type), warped to a mean lobe shape and overlaid, with a different colour used for clones from each petal. (F–J) Clones on ventral lobes (in which cyc and dich are inactive) from several div mutant flowers induced at a range of stages: (F) 300 h (day 12.5), (G) 340 h (day 14), (H) 380 h (day 16), (I) 420 h (day 17.5), and (J) 440 h (day 18) (note that developmental timing does not correlate perfectly with wild type), warped to a mean lobe shape and overlaid, with a different colour used for clones from each petal. (2.85 MB TIF) [file pbio.1000537.s013.tif]
