# Supplementary material for: Genetic Control of Organ Shape and Tissue Polarity
Source: PLoS Biol. 2010 Nov 9;8(11):e1000537. doi: 10.1371/journal.pbio.1000537 (PMC2976718; doi:10.1371/journal.pbio.1000537)
Supplement: Text S1 — (A) Data on clonal analysis. (B) Snapdragon model. (0.09 MB PDF) [file pbio.1000537.s016.pdf]

Text S1

A Data on clonal analysis including 13 figures and two tables

B Snapdragon Model (description and equations)

A Data on clonal analysis

Clones induced at the same developmental stage for different petals were overlaid using the Sector Analysis Toolbox software. Red sectors (clones) were segmented from the pale background. The sectors from each petal were coloured differently and warped to a calculated mean petal shape to allow comparison and overlay (necessary to achieve high enough sector density to observe patterns). This process was repeated for two independent sets of petals at each developmental stage (Fig. S1A-E; Fig. S2-11).

To extract growth data from the clone patterns, the method described in (Rolland-Lagan *et al.*, 2005) was applied, using the Sector Analysis Toolbox. The maximal growth rates for four $40h$ steps between $300h$ and $460h$ were visualised, as well as the orientation of the principal direction of growth (Fig. S1F-I). For each step, the grids used to divide the petals were optimised by applying a specified minimum number of clones per region for both stages. This meant that a slightly different grid was used for each step (Fig. S12).

Each sector was approximated with an ellipse. For each region and stage, the mean ellipse orientation (θ) with respect to a standard axis for the petal, and the mean length of the major and minor axes of the ellipses (E_{maj} and E_{min}) were calculated (Table S1). These data were used to determine the growth rates along the major (K_{max}) and minor (K_{min}) axes and the principal direction of growth (Table S2).

B Snapdragon Model

The detailed computational methods are described in "From genes to physical shape: a method for modelling growing tissues" (R. Kennaway *et al.*, in submission, 2010). Here we outline the interaction functions involved in each model. To simplify the functions two standard functions are provided to model promotion and inhibition of one factor by another. These are:

$$\begin{aligned}\text{pro}(k, \mathbf{x}) &= 1 + k\mathbf{x} \\ \text{inh}(k, \mathbf{x}) &= 1/(1 + k\mathbf{x})\end{aligned}$$

We use boldface for vectors of values, one value per mesh vertex, and italic for scalar values. Multiplication and division of vectors are to be understood elementwise. $\text{pro}(k, \mathbf{x})$ and $\text{inh}(k, \mathbf{x})$ both tend to vectors of ones as the components of $\mathbf{x}$ tend to zero. Identity and signalling factors are symbolised with i and s respectively, subscripted with the relevant name. Each model has three or four components: setup, PRN, (GRN), KRN.

Model 1: Ground state (*cyc dich div* triple mutant)

Setup

The setup provides an initial canvas together with a pattern of factors and is not part of the growth simulation that starts at 250 hrs. The initial canvas (Fig. 4D) is imported as a pre-defined mesh of finite elements. The pattern of identity factors is imposed according to the geometry of the mesh. Typically, the values of identity factors range from 0 (inactive) to 1 (fully active).

PRN

Polariser POL is first produced during the setup phase to establish proximodistal polarities prior to growth. The PROXORG identity factor is activated where PROX is above a threshold (Eqn. P.1), while DISTORG is activated by DIST but inhibited by LAT (Eqn. P2).
From time 230 generate polariser:

$$\mathbf{i}_{proxorg} = (\mathbf{i}_{prox} > 0.8) \qquad \text{P.1}$$

At time 230 hrs:

$$\mathbf{i}_{distorg} = 0.25 \cdot \mathbf{i}_{dist} \cdot \text{inh}(100, \mathbf{i}_{lat}) \qquad \text{P.2}$$

The diffusion, production, degradation and decay of POL is specified by

$$\partial \mathbf{s}_{pol}/\partial t = D_{\mathbf{s}_{pol}} \nabla^2 \mathbf{s}_{pol} + 0.1 \cdot (\mathbf{i}_{proxorg} + 1 - \mathbf{s}_{pol} \cdot \mathbf{i}_{distorg}) - a_{\mathbf{s}_{pol}} \mathbf{s}_{pol} \qquad \text{P.3}$$

where, $D_{\mathbf{s}_{pol}} = 0.002\ mm^2/hr$ is the effective diffusion constant for POL and $a_{\mathbf{s}_{pol}} = 0.1\ hr^{-1}$ is the decay term for POL. To maintain a more consistent POL gradient during the late phase of growth a gradient of DISTORG is established using a signalling factor $\mathbf{s}_{distorg}$. $\mathbf{s}_{distorg}$ is activated by DISTORG as follows:

$$\partial s_{distorg}/\partial t = D_{s_{distorg}} \nabla^2 s_{distorg} + 0.1 \cdot \mathbf{i}_{distorg} - a_{s_{distorg}} s_{distorg} \qquad \text{P.4}$$

where, $D_{\mathbf{s}_{distorg}} = 0.02\ mm^2/hr$ is the diffusion constant for $\mathbf{s}_{dist}$ and $a_{\mathbf{s}_{distorg}} = 0.05\ hr^{-1}$.
At the beginning of the late phase DISTORG is set to $\mathbf{s}_{distorg}$.
At time 340 hrs:

$$\mathbf{i}_{distorg} = \mathbf{s}_{distorg} \qquad \text{P.5}$$

KRN

The interactions described in Fig. 4F which set the values for K_{par} are captured mathematically in the following Eqn. (K.1).

$$K_{par} = \qquad \text{K.1}$$
$$0.013 \qquad \text{K.1.1}$$
$$\cdot\, \text{inh}(0.2, \mathbf{i}_{prox}) \qquad \text{K.1.2}$$
$$\cdot\, \text{pro}(0.4, \mathbf{i}_{tube} \cdot \text{inh}(100, \mathbf{i}_{plt})) \qquad \text{K.1.3}$$
$$\cdot\, \text{pro}(1.4, \mathbf{i}_{early} \cdot \mathbf{i}_{lip}) \qquad \text{K.1.4}$$
$$\cdot\, \text{inh}(10, \mathbf{i}_{early} \cdot \mathbf{i}_{rim}) \qquad \text{K.1.5}$$
$$\cdot\, \text{inh}(0.5, \mathbf{i}_{late} \cdot \mathbf{i}_{med} \cdot \mathbf{i}_{lobe}) \qquad \text{K.1.6}$$

The basic value of K_{par} is set to 0.013 (Term K.1.1). K_{par} is then modulated in various ways. For example, K_{par} is inhibited by a factor of 0.2 by $\mathbf{i}_{prox}$ (Term K.1.2), which is equivalent to multiplying by $1/(1 + 0.2\mathbf{i}_{prox})$ which equals 0.83 wherever $\mathbf{i}_{prox} = 1$. Similarly, K_{par} is promoted by a factor of 1.4 (Term K.1.4) by the combination of $\mathbf{i}_{early}$ and $\mathbf{i}_{lip}$ which is equivalent to multiplying by $1 + 1.4\mathbf{i}_{early} \cdot \mathbf{i}_{lip}$ which evaluates to 2.4 wherever both $\mathbf{i}_{early}$ and $\mathbf{i}_{lip}$ are equal to one. $\mathbf{i}_{early}$ has a value of one from 250 to 340 hours and is otherwise zero, while $\mathbf{i}_{late}$ has a value one from 340 hours onwards and is otherwise zero.

Likewise, the values for K_{per} are captured by Eqn. K.2.

$$K_{per} = \qquad \text{K.2}$$
$$0.0075 \qquad \text{K.2.1}$$
$$\cdot\, \text{inh}(0.2, \mathbf{i}_{prox}) \qquad \text{K.2.2}$$
$$\cdot\, \text{pro}(2, \mathbf{i}_{early} \cdot \mathbf{i}_{dist} \cdot \text{inh}(20, \mathbf{i}_{lat})) \qquad \text{K.2.3}$$
$$\cdot\, \text{pro}(1, \mathbf{i}_{late} \cdot \mathbf{i}_{dist}) \qquad \text{K.2.4}$$
$$\cdot\, \text{inh}(0.3, \mathbf{i}_{late} \cdot \mathbf{i}_{lobe} \cdot \mathbf{i}_{med}) \qquad \text{K.2.5}$$

growth rates on both surfaces (a and b) of the canvas are the same and are set according to K_{par} and K_{per}.

$$k^a_{par} = K_{par} \qquad \text{K.3}$$

$$k^b_{par} = K_{par} \qquad \text{K.4}$$

$$k^a_{per} = K_{per} \qquad \text{K.5}$$

$$k^b_{per} = K_{per} \qquad \text{K.6}$$

The rate of growth in thickness (k_{nor}) is set to 0.003 in accordance with experimental data.

$$k_{nor} = 0.003 \qquad \text{K.7}$$

Model 1, mutant 1: Ground state with no polariser

The interaction function is identical to that in Model 1 except that equation $P.3$ in the PRN is replaced by $P.3a$.

$$\partial \mathbf{s}_{pol}/\partial t = D_{\mathbf{s}_{pol}} \nabla^2 \mathbf{s}_{pol} + 0 - a_{\mathbf{s}_{pol}} \mathbf{s}_{pol} \qquad \text{P.3a}$$

In the absence of polariser specified growth is isotropic and takes the average value of K_{par} and K_{per}.

Model 1, mutant 2: Ground state without growth inhibited in the medial lobe

The interaction function is identical to that in Model 1 except that Terms $K.1.6$ and $K.2.5$ are omitted in the KRN.

Model 2: Incorporating DIV (*cyc dich* double mutant)

This model is the same as Model 1 with the following modifications.

DIV is activated during the setup phase with the following simple GRN: At time 240 hrs:

$\mathbf{i}_{div}$=1 G.1

The effects of DIV on the KRN outlined in Fig. 5E are as follows:

K_{par} = K.1

(as K.1.1...K.1.6)

$\cdot \, \text{pro}(2.4, \mathbf{i}_{early} \cdot \mathbf{i}_{div} \cdot \mathbf{i}_{plt})$ K.1.7

K_{per} = K.2

(as K.2.1...K.2.3, K.2.5)

$\cdot \, \text{pro}(1, \mathbf{i}_{late} \cdot \mathbf{i}_{dist} \cdot \text{pro}(1.2, \mathbf{i}_{div}))$ K.2.4a

$\cdot \, \text{inh}(1, \mathbf{i}_{late} \cdot \mathbf{i}_{div} \cdot \text{inh}(10, \mathbf{i}_{lobe}))$ K.2.6

k^{a}_{par} = K.3

(as K.3.1)

$+ \, 0.06 \cdot (\mathbf{i}_{early} \cdot \mathbf{i}_{rim}) \cdot (1.2 \cdot \mathbf{i}_{div})$ K.3.2

Model 3: First model of wild type

This model is the same as Model 1 with the following modifications. CYC and DICH are activated in two adjacent petals of the canvas during the setup phase (Fig. 6E). The GRN illustrated in Fig. 6D is implemented as follows:

GRN

At time 240 hrs:

$\mathbf{i}_{div} = \text{inh}(100, \mathbf{i}_{rad})$ G.1a

$\mathbf{i}_{lts}(:) = \mathbf{i}_{div}$ G.2

At time 250 hrs:

$\mathbf{i}_{cyc} = \mathbf{i}_{cyc} \cdot \text{inh}(4, \mathbf{i}_{div})$ G.3

$\mathbf{i}_{dich} = \mathbf{i}_{dich} \cdot \text{inh}(4, \mathbf{i}_{div})$ G.4

$\mathbf{i}_{lts} = 5 \cdot \mathbf{i}_{lts} \cdot \mathbf{s}_{rad}$ G.5

Always:

$\mathbf{i}_{rad} = \mathbf{i}_{cyc} + 0.3 \cdot \mathbf{i}_{dich}$ G.6

$\partial s_{rad}/\partial t \; = \; D_{s_{rad}} \nabla^2 s_{rad} \, + \, 0.1 \cdot \mathbf{i}_{rad} \cdot \text{pro}(0.5, \mathbf{i}_{tube}) \, - \, a_{s_{rad}} s_{rad}$ G.7

At time 340 hrs:

$\mathbf{i}_{div} = \mathbf{i}_{div} \cdot \text{inh}(5, \mathbf{i}_{lts})$ G.8

Changes in the KRN indicated in Fig. 6F are implemented as:

$$K_{par} = \quad \text{K.1}$$
(as K.1.1...K.1.3, K.1.5...K.1.7)
$$\cdot\, \mathrm{pro}(1.4, \mathbf{i}_{early} \cdot \mathbf{i}_{lip} \cdot \mathrm{inh}(100, \mathbf{i}_{rad} \cdot \mathrm{inh}(100, \mathbf{i}_{dich} \cdot \mathbf{i}_{lat}))) \quad \text{K.1.4a}$$
$$\cdot\, \mathrm{inh}(1, \mathbf{i}_{rad} \cdot \mathbf{i}_{lip} \cdot \mathrm{inh}(3, \mathbf{i}_{dich} \cdot \mathbf{i}_{lat})) \quad \text{K.1.8}$$
$$\cdot\, \mathrm{inh}(1, \mathbf{i}_{rad} \cdot \mathbf{i}_{plt} \cdot \mathrm{inh}(10, \mathbf{i}_{dich}) \cdot \mathrm{inh}(20, \mathbf{i}_{cyc} \cdot \mathrm{inh}(40, \mathbf{i}^2_{lat}))) \quad \text{K.1.9}$$
$$\cdot\, \mathrm{pro}(0.3, \mathbf{i}_{cyc} \cdot \mathbf{i}_{dtl} \cdot \mathrm{inh}(0.5, \mathbf{i}_{dich})) \quad \text{K.1.10}$$
$$\cdot\, \mathrm{pro}(0.45, (\mathbf{i}_{cyc} + 0.2 \cdot \mathbf{i}_{dich}) \cdot \mathbf{i}_{plt}) \quad \text{K.1.11}$$

$$K_{per} = \quad \text{K.2}$$
(as K.2.1...K.2.3, K.2.5, K.2.6)
$$\cdot\, \mathrm{pro}(1, \mathbf{i}_{late} \cdot \mathbf{i}_{dist} \cdot \mathrm{pro}(1.2, (\mathbf{i}_{cyc} + \mathbf{i}_{div}))) \quad \text{K.2.4b}$$
$$\cdot\, \mathrm{pro}(0.1, \mathbf{i}_{cyc} \cdot \mathrm{pro}(1.5, \mathbf{i}_{lip})) \quad \text{K.2.7}$$
$$\cdot\, \mathrm{inh}(6, \mathbf{i}_{early} \cdot \mathbf{i}_{lts} \cdot \mathbf{i}_{med}) \quad \text{K.2.8}$$
$$\cdot\, \mathrm{pro}(0.2, \mathbf{i}_{late} \cdot \mathbf{i}_{lts} \cdot \mathbf{i}_{med} \cdot \mathbf{i}_{plt}) \quad \text{K.2.9}$$

$$k^a_{par} = \quad \text{K.3}$$
(as K.3.1)
$$+\, 0.06 \cdot \mathbf{i}_{early} \cdot \mathbf{i}_{rim} \cdot (1.2 \cdot \mathbf{i}_{div} + 0.3 \cdot \mathbf{i}_{lts} \cdot \mathbf{i}_{med} + 0.5 \cdot (\mathbf{i}_{cyc} + \mathbf{i}_{dich})) \quad \text{K.3.2a}$$

Model 4: Promotion of perpendicular growth in the lateral petals

This model is the same as Model 3 except for the modification of the KRN shown in Fig. 7A which is implemented as

$$K_{per} = \quad \text{K.2}$$
(as K.2.1...K.2.9)
$$\cdot\, \mathrm{pro}(3.5, \mathbf{i}_{late} \cdot \mathbf{i}_{lts} \cdot \mathbf{i}_{med} \cdot \mathrm{inh}(4, \mathbf{i}_{lat}) \cdot \mathrm{pro}(1, (\mathbf{i}_{lip} + 0.3 \cdot \mathbf{i}_{plt}))) \quad \text{K.2.10}$$

Model 5: Introduction of a central organiser

This model is the same as Model 3 except for the following modifications. The changes to the PRN as shown in Fig. 7E are implemented by first activating CENORG with a combination of DIV, LAT and RIM:

$$\mathbf{i}_{cenorg} = 0.6 \cdot \mathbf{i}_{lat} \cdot \mathbf{i}_{rim} \cdot \mathbf{i}_{div} \quad \text{P.6}$$

The diffusion, production, degradation and decay of POL is specified by replacing Eqn. P.3 with P.3b

$$\partial \mathbf{s}_{pol}/\partial t = D_{\mathbf{s}_{pol}} \nabla^2 \mathbf{s}_{pol} + 0.1 \cdot (\mathbf{i}_{proxorg} + 1 - \mathbf{s}_{pol} \cdot (\mathbf{i}_{distorg} + 3 \cdot \mathbf{i}_{late} \cdot \mathbf{i}_{cenorg})) - a_{\mathbf{s}_{pol}} \mathbf{s}_{pol} \quad \text{P.3b}$$

To maintain a more consistent POL gradient, a gradient of CENORG is established using a signalling factor $\mathbf{s}_{cenorg}$. $\mathbf{s}_{cenorg}$ is activated by CENORG as follows:

$$\partial s_{cenorg}/\partial t = D_{s_{cenorg}} \nabla^2 s_{cenorg} + 0.1 \cdot \mathbf{i}_{cenorg} - a_{s_{cenorg}} s_{cenorg} \quad \text{P.7}$$

where, $D_{\mathbf{s}_{cenorg}} = 0.002\ mm^2/hr$ is the diffusion constant for $\mathbf{s}_{cenorg}$ and $a_{\mathbf{s}_{cenorg}} = 0.05\ hr^{-1}$.
At the beginning of the late phase CENORG is set to $\mathbf{s}_{cenorg}$.
At time 340 hrs:

$$\mathbf{i}_{cenorg} = \mathbf{s}_{cenorg} \qquad \text{P.8}$$

The KRN as indicated in Fig. 7F is implemented with:

K_{par} = K.1

(as K.1.1 . . . K.1.11)

$\cdot\, \text{pro}(2.2, \mathbf{i}_{late} \cdot \mathbf{i}_{lts} \cdot \mathbf{i}_{med} \cdot \text{inh}(0.5, \mathbf{i}_{lpb}) \cdot \text{inh}(4, \mathbf{i}_{lat}) \cdot (\mathbf{i}_{lip} + 0.3 \cdot \mathbf{i}_{plt}))$ K.1.12

Bending back of the petals was slightly modulated by reducing the multiplier in Term K.3.2a from 0.06 to 0.05:

k^a_{par} = K.3

(as K.3.1)

$+\, 0.05 \cdot \mathbf{i}_{early} \cdot \mathbf{i}_{rim} \cdot (1.2 \cdot \mathbf{i}_{div} + 0.3 \cdot \mathbf{i}_{lts} \cdot \mathbf{i}_{med} + 0.5 \cdot (\mathbf{i}_{cyc} + \mathbf{i}_{dich}))$ K.3.2b

Model 6: Wild type with improved shape

This model is the same as Model 5 except for modifications to the KRN shown in Fig. 8A implemented as follows:

K_{par} = K.1

(as K.1.1 . . . K.1.12)

$\cdot\, \text{inh}(10, \mathbf{i}_{late} \cdot \mathbf{i}_{div} \cdot \mathbf{i}_{cenorg} \cdot \text{inh}(2, \mathbf{i}_{lpb}) \cdot \text{pro}(10, \mathbf{i}_{plt} \cdot \mathbf{i}_{med}))$ K.1.13

$\cdot\, \text{pro}(0.2, \mathbf{i}_{late} \cdot \mathbf{i}_{div} \cdot \mathbf{i}_{plt})$ K.1.14

$\cdot\, \text{inh}(0.7, \mathbf{i}_{late} \cdot \mathbf{i}_{lts} \cdot \mathbf{i}_{lat} \cdot (\mathbf{i}_{lip} + \mathbf{i}_{plt}))$ K.1.15

K_{per} = K.2

(as K.2.1 . . . K.2.5, K.2.7 . . . K.2.9)

$\cdot\, \text{inh}(1.3, \mathbf{i}_{late} \cdot \mathbf{i}_{div} \cdot \text{inh}(2.5, \mathbf{i}_{lobe} \cdot \text{inh}(2, \mathbf{i}_{lpb})) \cdot \text{pro}(1, \mathbf{i}_{plt}))$ K.2.6a

k^a_{par} = K.3

(as K.3.1, K.3.2)

$\cdot\, \text{inh}(1, \mathbf{i}_{dich} \cdot \mathbf{i}_{lat} \cdot \mathbf{i}_{lpb})$ K.3.3

$\cdot\, \text{inh}(1, \mathbf{i}_{early} \cdot (\mathbf{i}_{cyc} + \mathbf{i}_{div}) \cdot \mathbf{i}_{mlobe} \cdot \text{inh}(5, \mathbf{i}_{dich}))$ K.3.4

$\cdot\, \text{pro}(1, \mathbf{i}_{early} \cdot \mathbf{i}_{div} \cdot \mathbf{i}_{lpb} \cdot \text{inh}(5, \mathbf{i}_{med}))$ K.3.5

k^b_{par} = K.4

(as K.4.1)

$\cdot\, \text{pro}(2, \mathbf{i}_{dich} \cdot \mathbf{i}_{lat} \cdot \mathbf{i}_{lpb})$ K.4.2

k^a_{per} = K.5

(as K.5.1)

$\cdot\, \text{pro}(1, \mathbf{i}_{early} \cdot \mathbf{i}_{div} \cdot \mathbf{i}_{lpb} \cdot \text{inh}(3, \mathbf{i}_{med}))$ K.5.2

k^b_{per} = K.6

(as K.6.1)

$\cdot\, \text{inh}(1, \mathbf{i}_{early} \cdot \mathbf{i}_{div} \cdot \mathbf{i}_{lpb} \cdot \text{inh}(3, \mathbf{i}_{med}))$ K.6.2

Model 7: Wild type with DICH influencing DISTORG

This model is the same as Model 6 except for changes in the PRN shown in Fig. 9A implemented during the setup phase with:
At time 230 hrs:

$$\mathbf{i}_{distorg} = 0.25 \cdot \mathbf{i}_{dist} \cdot \text{inh}(100, \mathbf{i}_{lat}) + 0.1 \cdot \mathbf{i}_{dich} \cdot \mathbf{i}_{dist} \cdot \mathbf{i}_{cyc} \qquad \text{P.2a}$$

Mutants of Model 7 are implemented by inactivating the relevant identity factors (e.g. CYC, DICH, DIV) by setting their values to zero.

Model 8: Wild type with CENORG slightly active in the ground state

This model is the same as Model 7 except for the following modifications. A basic level of CENORG is established in the PRN by replacing Eqn. P.6 with

$$\mathbf{i}_{cenorg} = \mathbf{i}_{lat} \cdot \mathbf{i}_{rim} \cdot (0.03 + 0.57 \cdot \mathbf{i}_{div}) \qquad \text{P.6a}$$

A basic level of bending back at the RIM is established in the KRN by replacing Term. K.3.2b with

$$+ 0.05 \cdot \mathbf{i}_{early} \cdot \mathbf{i}_{rim} \cdot (0.5 + 0.7 \cdot \mathbf{i}_{div} + 0.3 \cdot \mathbf{i}_{lts} \cdot \mathbf{i}_{med} + 0.5 \cdot \mathbf{i}_{dich}) \qquad \text{K.3.2c}$$
